# Transcriptome-enabled marker discovery and mapping of plastochron-related genes in *Petunia* spp.

**DOI:** 10.1186/s12864-015-1931-4

**Published:** 2015-09-24

**Authors:** Yufang Guo, Krystle E. Wiegert-Rininger, Veronica A. Vallejo, Cornelius S. Barry, Ryan M. Warner

**Affiliations:** Department of Horticulture, Michigan State University, East Lansing, MI 48824 USA

**Keywords:** *Petunia*, Transcriptome sequencing, Molecular markers, Single nucleotide polymorphisms, Development rate, Floriculture

## Abstract

**Background:**

Petunia (*Petunia × hybrida*), derived from a hybrid between *P. axillaris* and *P. integrifolia*, is one of the most economically important bedding plant crops and *Petunia* spp. serve as model systems for investigating the mechanisms underlying diverse mating systems and pollination syndromes. In addition, we have previously described genetic variation and quantitative trait loci (QTL) related to petunia development rate and morphology, which represent important breeding targets for the floriculture industry to improve crop production and performance. Despite the importance of petunia as a crop, the floriculture industry has been slow to adopt marker assisted selection to facilitate breeding strategies and there remains a limited availability of sequences and molecular markers from the genus compared to other economically important members of the Solanaceae family such as tomato, potato and pepper.

**Results:**

Here we report the *de novo* assembly, annotation and characterization of transcriptomes from *P. axillaris*, *P. exserta* and *P. integrifolia*. Each transcriptome assembly was derived from five tissue libraries (callus, 3-week old seedlings, shoot apices, flowers of mixed developmental stages, and trichomes). A total of 74,573, 54,913, and 104,739 assembled transcripts were recovered from *P. axillaris*, *P. exserta* and *P. integrifolia*, respectively and following removal of multiple isoforms, 32,994 *P. axillaris*, 30,225 *P. exserta*, and 33,540 *P. integrifolia* high quality representative transcripts were extracted for annotation and expression analysis. The transcriptome data was mined for single nucleotide polymorphisms (SNP) and simple sequence repeat (SSR) markers, yielding 89,007 high quality SNPs and 2949 SSRs, respectively. 15,701 SNPs were computationally converted into user-friendly cleaved amplified polymorphic sequence (CAPS) markers and a subset of SNP and CAPS markers were experimentally verified. CAPS markers developed from plastochron-related homologous transcripts from *P. axillaris* were mapped in an interspecific *Petunia* population and evaluated for co-localization with QTL for development rate.

**Conclusions:**

The high quality of the three *Petunia* spp. transcriptomes coupled with the utility of the SNP data will serve as a resource for further exploration of genetic diversity within the genus and will facilitate efforts to develop genetic and physical maps to aid the identification of QTL associated with traits of interest.

**Electronic supplementary material:**

The online version of this article (doi:10.1186/s12864-015-1931-4) contains supplementary material, which is available to authorized users.

## Background

The genus *Petunia* resides within the Solanaceae family and contains 20 species and subspecies that are native to South America [1]. *Petunia* × *hybrida* (petunia) is an important ornamental crop plant and represents a hybrid species derived in the nineteenth century from a cross between *P. axillaris* and *P. integrifolia* [2]. Subsequent breeding has introgressed traits from additional *Petunia* spp. that, together with natural variation resulting from mutations in key genes, have contributed to the wide diversity in plant and floral morphology and flower color that exists within the pool of commercially available germplasm [2–7]. In cool climates in the Northern Hemisphere, petunia is often produced in greenhouses during the winter months for distribution to spring markets once it reaches an optimal size and begins to flower [8, 9]. Therefore, a high percentage of the cost of crop production is related to energy consumption and growers are often faced with the dilemma of either reducing greenhouse temperatures, thereby extending the growing time of the crop and incurring increased labor costs, or elevating the growing temperature and increasing energy costs but reducing the duration of crop growth [8, 9]. Thus, understanding the factors that impact crop timing traits may facilitate the selection of petunia varieties with an increased rate of vegetative node formation (development rate) at either optimal or suboptimal growing temperatures, or varieties that initiate flowering following emergence of fewer leaf nodes. We have previously documented that accessions of *P. axillaris* and *P. integrifolia* possess increased development rate when compared to a diverse pool of commercial petunia germplasm, suggesting genetic variation for this trait within the genus [10]. This was confirmed in an interspecific F_2_ population of a cross between *P. axillaris* and *P. integrifolia* that identified three quantitative trait loci (QTL) on chromosomes 1, 2 and 5 that affected development rate and explained 34 % of the observed variation [11]. The molecular basis underlying these QTL remains to be identified.

The genetic determinants of development rate, often referred to as plastochron, are multifaceted, complex and not fully understood but are, at least in part, linked to hormonal control of meristem size and activity. For example, transgenic tobacco (*Nicotiana tabacum*) plants with increased cytokinin oxidase activity and a concomitant reduction in cytokinin levels displayed reduced meristem size and delayed plastochron when compared to wild type [12]. Similarly, characterization of Arabidopsis mutants with reduced auxin levels and disrupted auxin transport also influence plastochron [13–15]. In addition, mutations at the *plastochron 1* and *plastochron 2* loci of rice, which encode a cytochrome P450 of unknown function and a MEI2-like RNA binding protein homolog, respectively, also influence development rate but do so independently of each other [16, 17]. In Arabidopsis, the *SQUAMOSA PROMOTER BINDING PROTEIN-LIKE* (*SPL*) transcription factors *SPL9* and *SPL15* act redundantly to influence plastochron and over-expression of miR156, which targets multiple *SPL*s, shortens plastochron [18]. Analogous to the relationship of the *plastochron 1* and *2* loci of rice [17], the SPL/miR156 regulatory module acts independently of *CYP78A5/KLUH*, which is a putative ortholog of rice *plastochron 1* [18]. The involvement of the miRNA pathway in influencing plastochron is further supported by the characterization of the *serrate* and *altered meristem program* (*amp1*) mutants of Arabidopsis, which display reduced and increased rates of leaf initiation, respectively [19–22]. *SERRATE* encodes a zinc finger protein required for miRNA biogenesis and RNA splicing while AMP1 associates with ARGONAUTE1 at the endoplasmic reticulum and is required for translation inhibition through the exclusion of miRNA target mRNAs from polysomes [19, 20, 23]. Furthermore, mutations in *AMP1* homologs in maize and rice confer similar pleiotropic phenotypes to those exhibited by Arabidopsis *amp1* mutants, including altered plastochron [24, 25]. Together, these data suggest complex regulation of plastochron that involves different regulatory modules, including hormone and miRNA pathways.

The development of next generation sequencing technology has revolutionized biology and in particular, transcriptome sequencing provides a cost effective strategy for generating sequence and expression information from the gene space of non-model organisms or from species with large complex genomes [26, 27]. In plants, transcriptome sequencing has facilitated gene discovery, the development of molecular markers and large scale analyses of genetic variation [28–33]. Despite the economic and biological importance of petunia, genomic information and molecular marker resources for this genus are limited [11, 34–38], single nucleotide polymorphism (SNP) markers are currently unavailable and marker assisted selection is rarely utilized. In addition, although transcriptome resources are available for petunia, they are not extensive and most often are derived from the cultivated species *P. hybrida* or from specialized tissue types [39–41]. Herein, *de novo* assembly of reference transcriptomes of *P. axillaris*, *P. integrifolia* and *P. exserta*, derived from paired-end RNAseq analysis of five diverse tissues (callus, seedling, shoot apices, flowers and trichomes) is reported. Tissue types were selected to attempt to maximize the number of transcripts recovered while generating resources for studying traits of interest related to development and metabolism. These resources were utilized to develop a set of SNP, cleaved amplified polymorphic sequence (CAPS) and simple sequence repeat (SSR) markers that will facilitate QTL mapping and gene discovery for multiple traits within the genus, including those associated with development rate.

## Results and discussion

### Transcriptome assembly and annotation

Transcriptome sequencing of five tissue libraries, including callus, flowers, shoot apex, seedlings, and trichomes, from *P. axillaris*, *P. exserta*, and *P. integrifolia* yielded between ~248 and 294 M 100 nt reads, of which greater than 94 % passed quality and trimming filters (Table 1). A two-step *de novo* assembly strategy (see Additional file 1: Figure S1) modified from [42] resulted in 74,573, 54,913, and 104,739 transcripts ≥ 500 bp respectively, for *P. axillaris*, *P. exserta*, and *P. integrifolia*. The two-step assembly strategy was employed to eliminate redundant reads in the flower and trichome libraries already present in the callus, shoot apex, and seedling libraries to aid the quality of the assembly.

**Table 1:** Description of *Petunia* spp. tissues, libraries, and RNA-Seq data

Species	Tissue	Library	Number of raw reads (millions)	Number of filtered reads (millions)^a^
*P. axillaris*	Callus	AC	57.0	54.6 (95.8 %)
	Flower	AF	54.3	52.3 (96.2 %)
	Shoot Apex	AA	70.4	67.9 (96.3 %)
	Seedling	AS	65.3	62.8 (96.0 %)
	Trichome	AT	47.2	44.6 (94.5 %)
*P. exserta*	Callus	EC	36.8	34.8 (94.5 %)
	Flower	EF	66.7	63.0 (94.4 %)
	Shoot Apex	EA	56.6	53.6 (94.7 %)
	Seedling	ES	43.9	41.9 (95.3 %)
	Trichome	ET	45.0	41.6 (94.5 %)
*P. integrifolia*	Callus	IC	57.9	54.7 (94.5 %)
	Flower	IF	64.4	61.2 (95.0 %)
	Shoot Apex	IA	61.4	58.2 (94.8 %)
	Seedling	IS	50.2	47.5 (94.7 %)
	Trichome	IT	47.9	45.7 (95.4 %)

^a^The number and percentage of total raw reads included in each transcriptome assembly after quality filters were applied

**Fig. 1:**
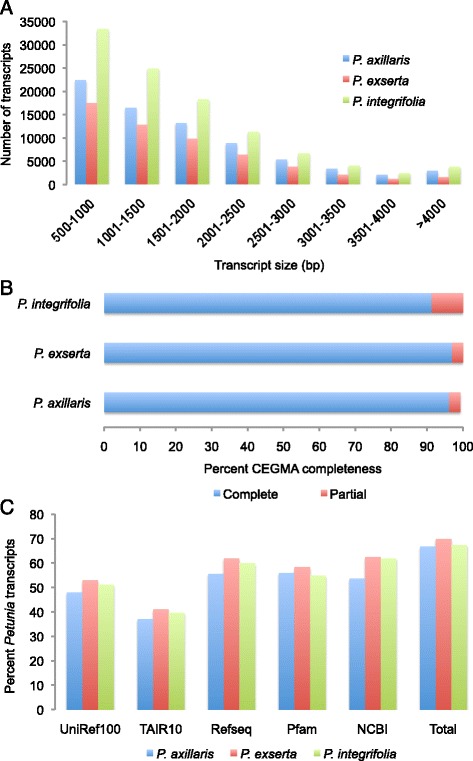
Quality metrics of the *Petunia* spp. transcriptome assemblies. **a** Size distribution of assembled transcripts. **b** CEGMA completeness assessment of the transcriptome assemblies. **c** Percentage of *P. axillaris*, *P. exserta*, and *P. integrifolia* unigenes with assigned functional annotations from UniRef100, TAIR10, RefSeq, the Pfam domain database, and NCBI GenBank non-redundant protein set. In addition, the total percentage of annotated unigenes per species is presented

The N50 value for each assembly was greater than 1950 and average transcript sizes of 1714 for *P. axillaris*, 1624 for *P. exserta*, and 1646 for *P. integrifolia* were obtained. The distribution of transcript sizes follows the same trend in each of the three species with the largest number of transcripts falling within the size bins of 500–1000 bp, and between 1001 and 1500 bp (Fig. 1a). CEGMA analysis [43] revealed that full length copies of >91 % of the highly conserved eukaryotic genes are present in each of the transcriptome assemblies while partial sequences are present for almost 100 % of these genes (Fig. 1b). These data are similar to those reported for other plant transcriptome assemblies [44, 45]. All high quality raw reads from each of the five libraries were also mapped back to their respective assembly with Bowtie and Tophat [46] generating mapping rates of 87.2 % *for P. axillaris*, 86.8 % for *P. exserta*, and 78.3 % for *P. integrifolia*.

For *P. axillaris* and *P. exserta*, the majority of representative transcripts comprised a single isoform whereas approximately 60 % of *P. integrifolia* transcripts possessed multiple isoforms (Table 2; Additional file 1: Figure S2). Where multiple isoforms were identified, the median number was two for *P. axillaris* and *P. exserta*, and three for *P. integrifolia*. The longest isoform was selected as the representative transcript yielding a total of 32,994, 30,225 and 33,540 representative transcripts in *P. axillaris*, *P. exserta*, and *P. integrifolia*, respectively. These transcripts covered between ~47.5 and 53.1 Mbp of the transcriptome space (Table 2). The increased number of transcripts retrieved from the *P. integrifolia* assembly together with a higher number of transcripts with multiple isoforms, is likely the result of widespread heterozygosity within this species due to self-incompatibility [47].

**Table 2:** Total number of *de novo* assembled transcripts in each of the *Petunia axillaris*, *P. exserta*, and *P. integrifolia* transcriptomes in relation to the number of representative transcripts and their total coverage of the transcriptome space

		Representative transcripts (no.)			
Species	Assembled transcripts (no.)	Single isoform	Multiple isoforms	Total	Transcriptome coverage (bp)
*P. axillaris*	74,573	18,487	14,507	32,994	53,135,953
*P. exserta*	54,913	19,517	10,708	30,225	47,589,955
*P. integrifolia*	104,739	13,009	20,531	33,540	49,087,745

Representative transcripts from each assembly were annotated using BLASTX searches against five publically available databases (Additional file 2: Dataset S1). Adopting quality thresholds of ≥30 % coverage and ≥70 % identity resulted in an annotation rate of approximately 70 % (Fig. 1c), with the largest number of annotations retrieved from the RefSeq and NCBI non-redundant databases. Similar rates of annotation were reported for transcriptome assemblies of chickpea and red clover [48, 49]. In addition, of the 1944 predicted *Petunia* proteins available in GenBank, ~89 % are present in the *P. axillaris*, *P. exserta*, and *P. integrifolia* transcriptome assemblies.

**Fig. 2:**
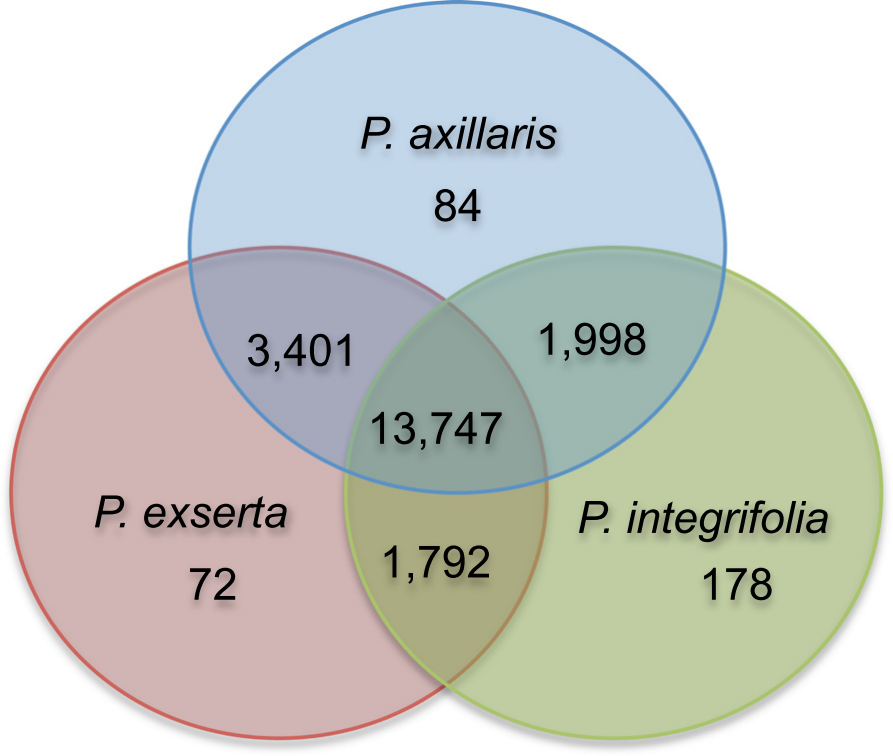
OrthoMCL identified orthologous gene clusters in the three *Petunia* species. A total of 21,272 orthologous clusters were identified among all comparisons. Unique and species specific OrthoMCL clusters are shown

**Fig. 3:**
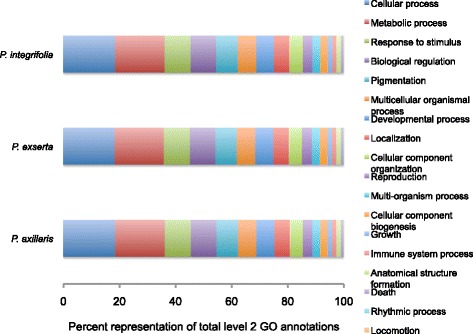
Biological Process GO annotation comparisons among species

Open reading frames (ORFs) were extracted from the representative transcripts and the predicted protein sequences were searched for orthologous gene clusters using OrthoMCL [50]. Among all comparisons, approximately 21,000 orthologous clusters were identified, with *P. axillaris*, *P. exserta*, and *P. integrifolia* sharing 13,747 clusters (Fig. 2; Additional file 3: Dataset S2). A larger number of orthologous clusters were found between *P. axillaris* and *P. exserta*, likely due to their closer phylogenetic relationship [1]. While *P. integrifolia* was found to house over double the number of unique clusters (Fig. 2; Supplemental Dataset 2). In addition, Gene Ontology (GO) annotations (Additional file 4: Dataset S3) of the representative transcripts showed highly equivalent representation of biological process categories among the three species, indicating the uniformity of the transcriptome assemblies and their subsequent annotation (Fig. 3).

### SNP detection and characterization

In the absence of whole genome sequences, comparative transcriptome analysis has proven utility for developing SNP markers [30, 31]. To facilitate genetic analysis within the genus, the three *Petunia* spp. transcriptomes were utilized for SNP discovery using the Genome Analysis Toolkit (GATK; see Additional file 1: Figure S3) [51]. When utilizing RNA-seq data for SNP discovery, removal of duplicate reads increases SNP detection sensitivity and specificity [52], thus this strategy was adopted resulting in utilization of 62.7 % of *P. axillaris*, 51.3 % of *P. exserta* and 60.8 % of *P. integrifolia* reads that uniquely mapped to the *P. axillaris* transcriptome (data not shown)*.* Depth of sequence coverage increases the reliability of SNP detection [52] and utilization of a minimum threshold for read coverage depth of 10 resulted in mapping of 93.7 %, 81.2 % and 73.3 % of the reads for *P. axillaris*, *P. exserta*, and *P. integrifolia*, respectively (Table 3).

**Table 3:** Summary of read depth coverage for *Petunia axillaris*, *P. exserta*, and *P. integrifolia* reads mapped to the *P. axillaris* transcriptome assembly after de-duplication

			Granular Quartile Read Depths			% of Reference Transcriptome with Read Depth Coverage:					
Sample	Total reads (millions)	Mean read depth	Third (25 %)	Median (50 %)	First (75 %)	>10	>15	>20	>30	>40	>50
*P. axillaris*	13.35	251	264	93	30	93.7	88.6	83.4	74.6	68.2	63.3
*P. exserta*	11.00	207	214	70	16	81.2	75.8	71.5	64.9	60.0	56.1
*P. integrifolia*	7.65	144	160	49	9	73.3	67.9	63.9	57.8	53.2	49.4
Total	32.00	355									

Overall, there were 814,408 SNPs between the three species. Of these, 105,645 were located in 5ʹ untranslated regions (UTRs), 481,289 were located within the coding sequence (CDS), and 217,178 were located in 3ʹUTRs. SNP frequency was calculated by dividing the total length of the reference transcriptome by the total number of SNPs. When only considering the length of 5ʹUTRs, CDS, and 3ʹUTRs in transcripts (not the length of the genomic regions these transcripts spanned), the SNP frequencies were 1 SNP/69 bp, 1 SNP/61 bp and 1 SNP/62 bp in these regions, respectively. The overall SNP frequency was 1 SNP/63 bp.

After filtering, among the 32,994 representative *P. axillaris* transcripts (unigenes), we identified SNPs between *P. axillaris* and either *P. exserta* or *P. integrifolia* in 20,606 unigenes. Gene Ontology (GO) annotation revealed that among all unigenes, 22,535 (68.3 %) contained GO terms, while 16,787 (80.8 %) of the SNP-containing unigenes were assigned with one or more GO ID (Additional file 1: Figure S4). In general, the distribution of GO terms was very similar between all unigenes and those containing SNPs. KEGG Pathway analysis was carried out to determine functional categorization of unigenes containing SNPs. A total of 5558 (27.8 % of total) unigenes containing SNPs were annotated by 2910 KO (KEGG Orthology) identifiers by using only references appropriate for plant species (Table 4). These unigenes were assigned to 322 KEGG pathways. Overall, the SNP frequency for genes involved in metabolism and organismal systems super pathways was lower than those involved in genetic information processing and environmental information processing.

**Table 4:** KAAS (KEGG Automatic Annotation Server) analysis of super pathways involving SNP-containing transcripts

Super pathways^a^	Annotation entries^b^	Number of genes	Number of SNPs
Metabolism			
Amino acid metabolism	422	322	1530
Biosynthesis of other secondary metabolites	157	117	449
Carbohydrate metabolism	702	449	2451
Energy metabolism	287	265	1136
Glycan biosynthesis and metabolism	129	95	470
Lipid metabolism	340	242	285
Metabolism of cofactors and vitamins	185	185	748
Metabolism of other amino acids	135	122	547
Metabolism of terpenoids and polyketides	135	134	630
Nucleotide metabolism	193	139	719
Xenobiotics biodegradation and metabolism	79	44	199
Genetic Information Processing			
Folding, sorting and degradation	455	418	2289
Replication and repair	240	139	914
Transcription	212	212	1289
Translation	492	458	2432
Environmental Information Processing			
Membrane transport	23	23	189
Signal transduction	970	504	2719
Signaling molecules and interaction	3	3	35
Cellular Processes			
Cellular community	118	63	373
Cell growth and death	351	204	1202
Cell motility	60	60	339
Transport and catabolism	288	266	1535
Organismal System			
Development	72	36	135
Immune system	324	158	811
Environmental adaptation	183	179	971

^a^Only pathways relevant to plants are reported in the table

^b^Annotation entry is the unique record of the transcript (gene) name and gene Ko number; multiple genes may have the same Ko number

**Fig. 4:**
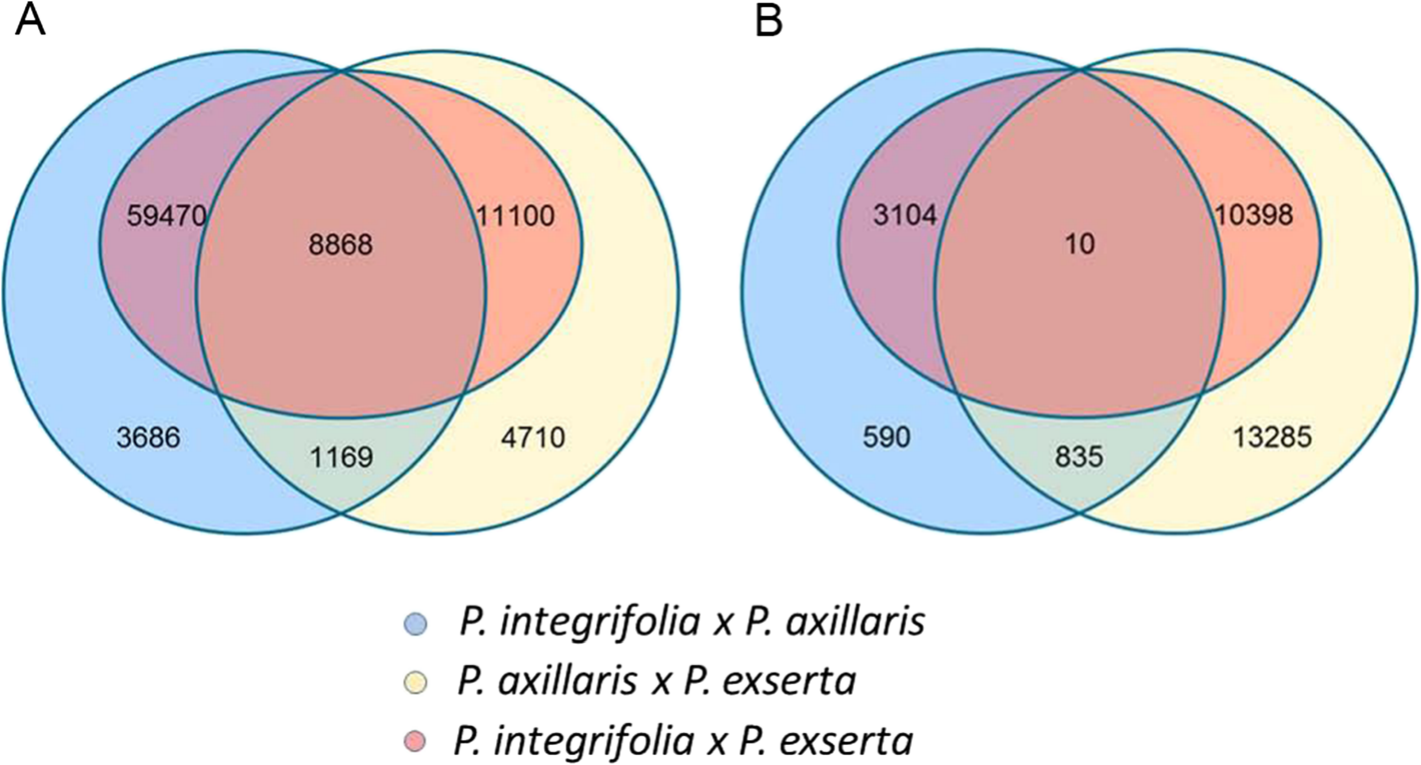
Summary of SNP loci between three *Petunia* spp. **a** Total number of SNP loci between P. *axillaris*, *P. exserta* and *P. integrifolia*; **b** number of homozygous SNP loci between P. *axillaris*, *P. exserta* and *P. integrifolia*. The intersecting portions of the Venn diagram illustrate the number of common loci between the comparisons

In total, 89,007 SNP positions were detected among *P. axillaris*, *P. exserta* and *P. integrifolia*. Of these, 8868 (10.0 %) were polymorphic across all three species combinations. This calculation was based on positions for which at least one species, *P. integrifolia* or *P. exserta,* had sufficient depth of coverage (10X). After removing low depth of coverage SNPs, 73,193 SNPs remained between *P. axillaris* and *P. integrifolia*, 25,847 SNPs remained between *P. axillaris* and *P. exserta*, and 79,438 SNPs remained between *P. exserta* and *P. integrifolia*. (Fig. 4a).

SNPs were further classified based on their location; CDS or UTR and their zygosity. Altogether, only 10 homozygous SNPs were polymorphic across all three species combinations (Fig. 4b). Between *P. axillaris* and *P. exserta*, 24,528 SNPs were homozygous, which comprised 94.9 % of all SNPs. There were 13,512 homozygous SNPs, 17 % of the total SNP positions, between *P. exserta* and *P. integrifolia*. Between *P. integrifolia* and *P. axillaris*, there were only 4539 homozygous SNPs, which constituted only 6.2 % of all polymorphisms, indicating that the self-incompatible *P. integrifolia* is highly heterozygous. Of all SNP loci, 60,701 were located within the CDS, of which 60,561 were biallelic; 9537 were located within 5ʹUTRs, of which 9512 were biallelic; 17,983 were located within 3ʹUTRs, of which 17,923 were biallelic. There is a small group of 786 SNPs whose location could not be determined.

As with the unfiltered SNPs, SNP frequency was calculated by dividing the total length of the reference transcriptome (Table 2) by the total number of SNPs (Table 5). Overall, the SNP frequency was 1/597 bp. The SNP frequency was 1/2056 bp between *P. axillaris* and *P. exserta*, 1/726 bp between *P. axillaris* and *P. integrifolia*, and 1/669 bp between *P. integrifolia* and *P. exserta*. All SNPs among the three species were distributed across 20,606 unigenes (62.5 % of the total unigenes), corresponding to ~76 % (40,556,099/53,135,953 bp) of the entire unigene length. When only considering the unigenes containing SNPs, the overall SNP frequency was 1 SNP/456 bp (89,007 SNPs/40,556,099 bp) and the highest SNP frequency was 1/89 bp. Among the SNP-containing unigenes, only 1944 (9.4 %) had 10 or more SNPs (Table 5). This suggests that the SNPs were broadly distributed across the transcriptome, which might facilitate SNP marker selection for genome wide association (GWAS) studies.

**Table 5:** SNP frequency, the number of unigenes which contain SNPs, and percentage of the total unigene length for all three species combinations

Species Comparison	SNP frequency	SNP-containing unigenes (no.)	Total unigene length containing SNPs (%)	Unigenes with ≥10 SNPs (no.)	SNP-containing unigenes with ≥10 SNPs (%)	Maximum SNP frequency per unigene
Overall	1/597 bp	20606	76	1944	9.42	1/89 bp
*P. axillaris* and *P. exserta*	1/2056 bp	12060	49	110	0.91	1/111 bp
*P. axillaris* and *P. integrifolia*	1/726 bp	17949	70	1466	8.17	1/96 bp
*P. exserta *and P. integrifolia	1/669 bp	18032	70	1722	9.55	1/89 bp

SNPs between *P. axillaris* and *P. exserta* were distributed across 12,060 unigenes (Table 5). There were only 110 unigenes with 10 or more SNPs. The maximum SNP frequency per unigene was 1 SNP/111 bp. Between *P. axillaris* and *P. integrifolia*, SNPs were identified in 17,949 unigenes. There were 1466 unigenes with 10 or more SNPs. The maximum SNP frequency per unigene was 1 SNP/96 bp. There were 18,032 unigenes with SNPs between *P. integrifolia* and *P. exserta*, and 1722 unigenes with more than 10 SNPs. The maximum SNP frequency per unigene was 1/89 bp.

Due to the stringent nature of the SNP discovery parameters employed (i.e., filtering out three SNPs occurring within 100 bp of each other and filtering out SNPs within the first and last of 30 bp of a transcript, etc.), the above SNP frequencies calculated after the filtering steps are likely underestimated. Additionally, for organisms without a fully sequenced genome, using a transcriptome *de novo* assembly followed by variant detection can result in underestimation of expressed variants [52]. Even with the collection of different tissue types in our study, there are still likely to be undetected variants.

**Fig. 5:**
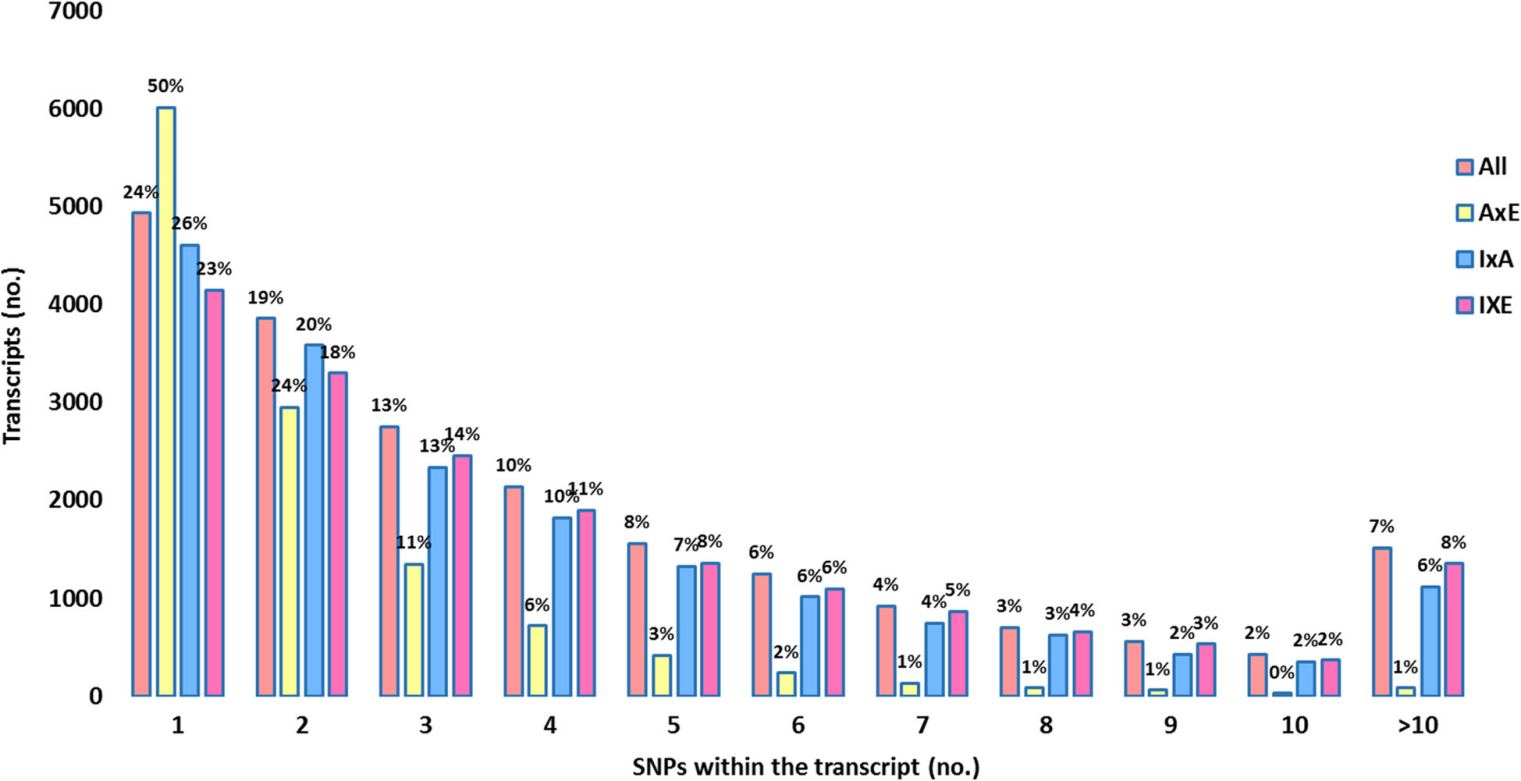
Distribution of transcripts with different number of SNPs. The x-axis indicates the number of SNPs within each transcript and the y-axis indicates the number of transcripts within the category. The percentage on top of each bar indicates the percentage of total transcripts fall into the category. All – all three species, A × E (*P. axillaris* × *P. exserta*), I × A (*P. integrifolia* × *P. axillaris*), I × E (*P. integrifolia* × *P. exserta*)

The percentage of unigenes with ten or more SNPs was 0.9 %, 8.2 % and 7.5 % of the entire SNP-containing unigene set between *P. axillaris* and *P. exserta*, *P. axillaris* and *P. integrifolia*, and *P. integrifolia* and *P. exserta*, respectively (Fig. 5). On average, there were 2.7 SNPs/transcript, with 2.2, 0.78 and 2.4 SNPs/transcript between *P. axillaris* and *P. integrifolia*, *P. axillaris* and *P. exserta*, and *P. integrifolia* and *P. exserta*, respectively*.* When evaluating the transcripts with more than ten SNPs for GO annotation, the gene percentage in each category was in proportion with the overall transcriptome GO annotation (Additional file 1: Figure S5), indicating no particular set of genes were enriched regarding SNP frequency.

To gain additional insight into base substitutions occurring at each polymorphic site, the transition to transversion ratios (Ts/Tv) were determined (Table 6). Overall, the transition to transversion ratio (Ts/Tv) was 1.7:1. Across all species comparisons, the Ts/Tv was consistently highest for SNPs within CDS, followed by SNPs located in 3ʹUTRs, while the Ts/Tv was always lowest for SNPs located in 5ʹUTR. The overall Ts/Tv was relatively stable between species, with a range of 1.6 to 1.8 (Table 6). The Ts/Tv ratio was used as a critical metric for assessing the specificity of new SNP calls in human genome research [53], and might be a useful parameter for subsequent *Petunia* SNP discovery. Normally, assuming that mutations are completely random, the Ts/Tv would be 0.5. Our Ts/Tv data indicate that each type of transitional change is produced more than three times as often as each type of transversion. Transitions occurring more frequently than transversions in transcriptome-derived SNPs have also been reported in other plant species, including Chinese pine [54] and melon [55]. The Ts/Tv bias could be the result of a high degree of methyl C to U in genomes [56, 57]. In plants, Ts/Tv can vary across species. For example, in the exome assembly of four Neotropical tree species, Ts/Tv varied between 1.5 and 1.7 [58]. In the transcriptome from Norway spruce, the Ts/Tv was around 1.2 to 1.5 depending on the sequencing quality cut-offs [59]. It has been suggested that a higher C↔T transition can be accompanied by a higher number of Ts. The same situation was observed in our study. For instance, SNPs between *P. integrifolia* and *P. axillaris*, and between *P. exserta* and *P. integrifolia*, had a higher rate of C/T mutations than was observed between *P. axillaris* and *P. exserta*, which was accompanied by a slightly higher Ts/Tv.

**Table 6:** SNP distribution and corresponding transition to transversion ratio (Ts/Tv) in coding regions (CDS), 5′UTRs and 3′UTRs

	Number	%	A/G Transition	C/T Transition	T/G Transversion	A/C Transversion	A/T Transversion	C/G Transversion	Ts/Tv
All SNPs (biallelic)	88,730	99.7 %^a^	31.5 %	32.0 %	8.4 %	8.5 %	12.1 %	7.6 %	1.7
SNPs in CDs (biallelic)	60,511		32.4 %	33.2 %	7.9 %	8.1 %	11.3 %	7.0 %	1.9
SNPs in 5’UTRs (biallelic)	9512		28.1 %	29.3 %	9.5 %	9.6 %	14.1 %	9.3 %	1.4
SNPs in 3′UTRs (biallelic)	17,923		30.2 %	29.4 %	9.5 %	8.9 %	13.6 %	8.5 %	1.5
*P. axillaris* and *P. exserta (biallelic)*	25,650	99.2 %	30.5 %	31.2 %	8.9 %	9.1 %	12.2 %	8.0 %	1.6
SNPs in CDs (biallelic)	14,686	99.1 %	31.7 %	32.9 %	8.4 %	8.8 %	11.0 %	7.2 %	1.8
SNPs in 5′UTRs (biallelic)	3842	99.5 %	27.6 %	29.1 %	10.0 %	10.2 %	13.9 %	9.2 %	1.3
SNPs in 3′UTRs (biallelic)	6753	99.3 %	29.7 %	28.9 %	9.6 %	9.0 %	14.0 %	8.8 %	1.4
*P. integrifolia* and *P. axillaris (biallelic)*	72,926	99.6 %	31.8 %	32.3 %	8.2 %	8.3 %	12.0 %	7.5 %	1.8
SNPs in CDs (biallelic)	52,413	99.7 %	32.6 %	33.3 %	7.8 %	8.0 %	11.3 %	7.0 %	1.9
SNPs in 5′UTRs (biallelic)	6765	99.7 %	28.7 %	29.5 %	9.0 %	9.4 %	14.2 %	9.3 %	1.4
SNPs in 3′UTRs (biallelic)	13,263	99.6 %	30.3 %	29.6 %	9.5 %	8.9 %	13.5 %	8.3 %	1.5
*P. exserta* and *P. integrifolia (biallelic)*	79,182	99.%	31.8 %	32.3 %	8.3 %	8.3 %	11.9 %	7.4 %	1.8
SNPs in CDs (biallelic)	56,820	99.7 %	32.7 %	33.3 %	7.9 %	8.0 %	11.2 %	6.9 %	1.9
SNPs in 5′UTRs (biallelic)	7396	99.7 %	28.2 %	29.8 %	9.4 %	9.3 %	14.0 %	9.3 %	1.4
SNPs in 3′UTRs (biallelic)	14,451	99.6 %	30.2 %	29.6 %	9.5 %	8.8 %	13.6 %	8.3 %	1.5

^a^Percentage was calculated based on the total SNP numbers

**Fig. 6:**
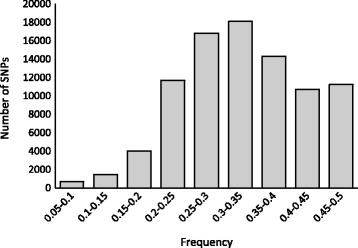
Minor allele reads count frequency distribution for SNPs among *P. axillaris*, *P. exserta*, and *P. integrifolia*

Minor allele read count frequency was used to calculate the frequency of short reads aligned to the least common allele in all three genotypes. Using a small number of genotypes for SNP discovery, this measure should provide better support for SNP confidence than using minor allele frequency (MAF), where the least prevalent allele frequency is calculated based on the genotype of each individual in a given population. For example, if there are only a few individuals in the panel, the smallest minor allele frequency will be 1/2n (n is the number of individuals), in our case, the minimum MAF would be 16.7 %. However, the average depth of coverage was above 100, and for RNA-seq data, the preferentially expressed genes will have a much higher read depth. Thus, even employing a depth cut-off value of 10, a heterozygous genotype call could have one allele with a much higher read count than the other allele, which might actually be caused by sequencing error or misalignment. This can be detected using the minor allele read count frequency. SNPs with minor allele reads count frequency ranging from 0.3 to 0.5 accounted for 61 % of the total SNPs (Fig. 6).

### SNP validation

A total of 55 primer pairs were selected for PCR amplification. Of these, 50 amplified single DNA fragments, containing a total of 51 SNP loci, matching the expected sizes in *P. axillaris* and were selected for further sequencing (Additional file 1: Table S1). The failed/unclear amplification might be from primers located across intron-exon boundaries, or from the amplification being across a large intron or in paralogous genes. Three of the loci were monomorphic, five yielded poor sequence quality, which was associated with either failure of the sequence reaction or ambiguous base calls that were likely due to heterozygosity of the parents that resulted in amplification of distinct alleles of a given locus. The remaining 43 loci were polymorphic between at least two species resulting in an overall SNP validation rate of 84.3 % (43/51). For the three monomorphic SNP loci, the minor allele sequence read count frequencies were 0.07, 0.26 and 0.33, indicating that low minor allele sequence read count frequency might be one factor that can introduce false positives in SNP identification. The validation rate obtained in this study is comparable with previously reported results for transcriptome derived SNPs. For example, the validation rate for pea (*Pisum sativum*) transcriptome derived SNPs was 84.5 % by selecting a 1920 SNPs set in a GoldenGate VeraCode assay on a large number of pea accessions [60]. The validation rate for the SNP detected from Douglas-fir (*Pseudotsuga menziesii*) was 72.5 % [61] while, in potato, the validation rate was 85 % for set of 96 transcriptome derived SNPs [62].

### CAPS marker design and validation

Twenty restriction enzymes were used to computationally predict CAPS markers from 15,701 SNP loci (Additional file 5: Dataset S4). Some SNP loci are predicted to be digested by two different restriction enzymes, with one recognizing the reference allele and the other recognizing the alternative allele, or by different restriction enzymes with overlapping recognition sequences. For example, for the restriction enzymes *Alu*I and *Sac*I, *Sac*I is a 6 bp cutter recognizing/cutting sequences of GAGCTˇC and *Alu*I is a 4 bp cutter that recognizes/cuts AGˇCT, thus all *Alu*I recognition cites are also recognized by *Sac*I. These analyses led to the identification of a total of 17,635 predicted CAPS markers (Supplemental dataset 4). Fourteen putative CAPS markers were randomly selected for validation. All primer pairs amplified products of the expected size, and 11 were polymorphic. All 14 amplicons for *P. axillaris* and *P. exserta* had the expected genotype, while four amplicons from *P. integrifolia* were homozygous while the genotype prediction was heterozygous. However, the CAPS markers generated from this study have some limitations. First, the CAPS marker design pipeline only considered possible restriction enzyme recognition differences between species, the number/size of the fragments generated from digestion was not taken into consideration. Thus, there might be multiple small fragments yielded from the digestion, or the digested fragments between genotypes may only vary by a few base pairs. Either situation could not be scored successfully on agarose gel genotyping systems. Second, there could be sequence differences between the *P. axillaris* reference genome and the actual parental genotype used for this study (a different *P. axillaris* accession), and some restriction enzyme recognition sites predicted in the pipeline might not exist when screening our samples.

### Simple sequence repeat (SSR) marker development

Of the 32,994 *P. axillaris* unigenes, 3027 (~9 %) contain predicted SSRs, and 375 (~12 %) of these possess more than one SSR (Table 7). The SSR frequency in the *P. axillaris* transcriptome is approximately 1 SSR per 15.2 kb, which is one-tenth of the SNP frequency between *P. axillaris*, *P. integrifolia* and *P. exserta*. The SSR frequency in *P. axillaris* is lower than the frequency in cassava (*Manihot esculenta*; 7.0 kb) [63], coffee (*Coffea* spp.; 7.7 kb) [64] and peanut (*Arachis hypogaea*) (5 kb) [65]. Among the *P. axillaris* SSRs, ~42 % are di-nucleotide repeats, of which AG/CT is most prevalent (43 %) and ~53 % are tri-nucleotide repeats with AAG/CTT repeats present at the highest frequency (~17.5 %; Table 8).

**Table 7:** Summary of SSR markers in the *P. axillaris* transcriptome

Total number of sequences examined	32,994
Total size of examined sequences (bp)	53,135,953
Total number of identified SSRs	3499
Number of SSR containing sequences	3027
Number of sequences containing more than 1 SSR	375
Number of SSRs present in compound formation	206
Di-nucleotide	1481
Tri-nucleotide	1867
Tetra-nucleotide	88
Penta-nucleotide	16
Hexa-nucleotide	47

**Table 8:** Summary of SSR repeat motif types and their corresponding repeat unit numbers for di- and tri- nucleotide repeats

Repeats	5	6	7	8	9	10	11	12	13	14	15	16	17	18	19	20	Total
AC/GT	-	203	132	76	56	38	15	15	6	7	7	4	2	2	1		564
AG/CT	-	209	130	81	40	34	25	17	17	14	13	10	4	5	9	3	611
AT/TA	-	122	64	29	19	10	9	8	7	3	2	1	1			1	276
AAC/GTT	186	72	23	17	8	5	4	4	4	1	1			1		2	328
AAG/CTT	205	68	35	11	6	6	7	4	3		2	1	6	3	1	3	361
AAT/ATT	107	47	24	6	3	3	3		1	1					2	2	199
ACC/GGT	159	56	21	4	1	1											242
ACG/CGT	7	2	1		1												11
ACT/AGT	77	24	7	11	4			1	1						1	1	127
AGC/CTG	104	33	15	9	7	1	2						1				172
AGG/CCT	72	17	7	7	3	1	1										108
ATC/ATG	182	68	17	8	10	3	1	1		1							291
CCG/CGG	19	2	1	1													23

A total of 2949 primer pairs were developed from 3027 unigenes (Additional file 6: Dataset S5). The designed SSR primer pairs included 1,481 di-, 1,867 tri-, 88 tetra-, 16 penta-, and 47 hexa- nucleotide repeats (Table 7). Forty-eight SSR markers were randomly selected for their utility in petunia (Additional file 7: Dataset S6). Of these, 36 (75 %) were successfully amplified and displayed polymorphisms between at least two of the three species examined. For example, 24 SSRs were polymorphic between *P. integrifolia* and *P. axillaris*, 28 were polymorphic between *P. axillaris and P. exserta*, and 24 were polymorphic between *P. integrifolia* and *P. exserta.* This success rate was higher than for our previous results developing SSR markers from *P. axillaris* ESTs [66].

### Representation and *in silico* expression analysis of *Petunia* plastochron-related transcripts

Several genes from Arabidopsis are known to influence plastochron (Table 9). Local tBLASTN searches of the *Petunia* spp. transcriptomes using 14 Arabidopsis plastochron-related proteins as the query sequences revealed the presence of highly homologous unigenes within each transcriptome (Table 9). The majority (34 out of 46) of the recovered transcripts are predicted to encode full-length proteins despite the fact that many plastochron-related genes, including *ERECTA*, *ERECTA LIKE-1*, *SERRATE* and *SLOW MOTION*, yield transcripts close to 3 kb or greater. Based on predicted amino sequence alignments, an additional five unigenes appear to be truncated by approximately 60 nucleotides (20 amino acids) or less at their N-termini (Table 9). This targeted analysis supports the transcriptome wide assessment of quality using CEGMA analysis (Fig. 1c) and indicates an overall high quality assembly of each transcriptome. Several of the previously characterized plastochron-related genes are preferentially expressed within the shoot apical meristem or developing leaf primordia [16, 18, 20]. Congruent with these previous findings, among the plastochron-related homologs identified in petunia, those related to *AMP1*, *ERECTA*, *PIN1*, *TEL1*, *KLUH*, *SERRATE* and *SPL15* display enriched expression within shoot apices compared to the additional tissues examined (Table 9). These data suggest conservation of both gene content and expression pattern of plastochron-related genes between petunia and Arabidopsis.

**Table 9:** Petunia homologs of plastochron-related genes from Arabidopsis

			Tissue type			
Unigene identifier	Annotation	Coverage^a^	Flower	Shoot apices	Seedlings	Trichomes
Paxi_locus_37_Transcript_7/9_Length_3129^b^	AMP1^c^	++	16.7^e^	41.8	30.1	13.6
Pexs_locus_18730_Transcript_7/8_Length_2607	AMP1	++	23.4	46.4	14.2	32.6
Pint_locus_21727_Transcript_1/5_Length_1316	AMP1^d^	-	6.2	21.9	8.9	3.8
Pint_locus_22203_Transcript_1/2_Length_1109	AMP1	-	5.1	13.8	8.9	3.4
Paxi_locus_7774_Transcript_1/1_Length_3771	SLOMO	++	26.7	22.2	23.7	20.3
Pexs_locus_3846_Transcript_1/2_Length_3848	SLOMO	++	29.7	24.2	19.9	25.6
Pint_locus_6038_Transcript_3/3_Length_3716	SLOMO	++	28.1	31.6	26	25.4
Paxi_locus_28305_Transcript_3/3_Length_4072	ER	++	33.9	81.5	22.2	29.4
Pexs_locus_27971_Transcript_3/3_Length_4072	ER	++	22.8	55.5	8.0	66.5
Pint_locus_23938_Transcript_2/6_Length_3741	ER	++	21.7	113	35.3	37.3
Paxi_locus_17737_Transcript_3/3_Length_3550	ERL1	++	13.1	33.1	4.3	62.8
Pexs_locus_28063_Transcript_1/2_Length_3819	ERL1	-	7.8	16	1.3	54.8
Pint_locus_64973_Transcript_1/10_Length_3648	ERL1	++	5.4	33	6.9	118.8
Paxi_locus_17054_Transcript_3/3_Length_2365	PIN1	++	26.3	42.8	17.3	14.1
Pexs_locus_18753_Transcript_1/1_Length_2550	PIN1	++	16.7	40.5	11.6	27.3
Pint_locus_7020_Transcript_2/7_Length_2155	PIN1	+	29	51.5	35.9	12
Paxi_locus_4307_Transcript_1/3_ Length_2763	PIN3	++	28.5	13.8	24.6	23.3
Pexs_locus_32081_Transcript_2/3_Length_2800	PIN3	++	23.0	20.8	17.1	19.0
Pint_locus_15123_Transcript_1/7_Length_3107	PIN3	++	56.6	35.6	146.5	27.8
Paxi_locus_2898_Transcript_1/1_Length_2484	TEL1	++	0.6	11	0.5	2.5
Pexs_locus_31850_Transcript_1/1_Length_2212	TEL1	+	0	9.7	0.8	10.8
Pint_locus_19236_Transcript_1/3_Length_2722	TEL1	++	0	9.3	0.9	2.0
Paxi_locus_11865_Transcript_1/1_Length_1775	KLU	++	5.7	46.1	11.2	6.6
Pexs_locus_32423_Transcript_1/1_Length_2010	KLU	+	3.2	29.2	5.4	17.8
Pint_locus_55018_Transcript_1/1_Length_1785	KLU	+	4.0	52.5	13.1	8.6
Paxi_locus_2128_Transcript_1/2_Length_3063	SER^f^	++	41.3	84.5	53.5	51.4
Pexs_locus_1424_Transcript_1/1_Length_2854	SER	++	31.7	63.8	31.5	54.2
Pint_locus_294_Transcript_1/3_Length_2067	SER	-	30.7	78.5	43.9	38
Paxi_locus_23917_Transcript_1/1_Length_2956	SER	-	1.7	9.2	2.8	2.2
Pexs_locus_14709Transcript_1/1_Length_2964	SER	++	1.8	10	1.8	7.6
Pint_locus_12396_Transcript_1/3_Length_2121	SER	-	2.4	19.1	4.3	5.4
Paxi_locus_1735_Transcript_5/6_Length_4646	SPL1	++	77.4	37.3	43.8	44.9
Pexs_locus_1184_Transcript_1/1_Length_3442	SPL1	++	17.7	21.8	8.2	18.2
Pint_locus_16_Transcript_3/5_Length_3091	SPL1	++	76.5	64.3	51.4	65.8
Paxi_locus_34631_Transcript_2/2_Length_962	SPL3	++	14.7	8.2	1.9	5.2
Pexs_locus_31900_Transcript_1/1_Length_1186	SPL3	-	294.9	110.5	220.0	183.8
Pint_locus_74351_Transcript_1/6_Length_1207	SPL3	-	21.1	32.1	4.5	14.9
Paxi_locus_11501_Transcript_1/4 _Length_1639	SPL9	++	36.5	33.1	3.9	11.9
Pexs_locus_21068_Transcript_1/2_Length_1577	SPL9	++	26.4	33.2	2.4	49.7
Pint_locus_21057_Transcript_2/3_Length_1378	SPL9	++	22.7	42.7	3.5	9.0
Paxi_locus_30395_Transcript_1/2_Length_1427	SPL15	++	3.5	34.1	1.4	3.5
Pexs_locus_31561_Transcript_1/1_Length_1265	SPL15	++	3.1	20.5	1.2	19.8
Pint_locus_74440_Transcript_1/2_Length_1316	SPL15	++	2.3	28.2	1.3	3.4
Pax_locus_6323_Transcript_2/3_Length_3369	ML5	++	6.5	10.2	8.0	8.6
Pexs_locus_16730_Transcript_1/2_Length_3261	ML5	++	7.1	10.7	5.7	11.2
Pint_locus_1362_Transcript_3/3_Length_3267	ML5	++	6.5	13.1	8.4	9.7

^a^Coverage refers to whether a unigene encodes a predicted full-length protein (++), is truncated by ~ 20 amino acids or less (+), or is truncated by greater than 20 amino acids (−)

^b^The designation Paxi, Pexs and Pint refer to unigenes derived from *P. axillaris*, *P. exserta* and *P. integrifolia*, respectively

^c^Gene symbols are as follows: *ALTERED MERISTEM PROGRAM 1* (*AMP1*), *SLOW MOTION* (*SLOMO*), *ERECTA* (*ER*), *ERECTA LIKE 1* (*ERL1*), *PIN FORMED 1* (*PIN1*), *TERMINAL EAR LIKE 1* (*TEL1*), *KLUH* (*KLU*), *SERRATE* (*SER*), *SQUAMOSA PROMOTER BINDING PROTEIN-LIKE* (*SPL*), *PIN FORMED 3 (PIN3), MEI2-LIKE PROTEIN 5* (*ML5*)

^d^Two fragmented unigenes corresponding to *AMP1* are present within the *P. integrifolia* transcriptome

^e^Transcript abundance was determined by RNAseq and data is presented as Fragments Per Kilobase of transcript per Million mapped reads

^f^Two paralogs with homology to *SERRATE* are present within each Petunia transcriptome

### Mapping plastochron-related homologous transcripts in an interspecific *Petunia* population

We identified *Petunia* transcripts homologous to numerous plastochron-related genes from *Arabidopsis* (Table 9). With the previously identified SNPs, 13 of these transcripts were converted to CAPS markers and used for linkage mapping (Additional file 1: Table S2). Together with the previously published SSR and CAPS markers [11], we constructed a linkage map with a total length of 289 cM consisting of 90 markers. Of those, eight were CAPS markers developed from plastochron-related transcripts located on five chromosomes (1, 2, 3, 6, and 7). The average linkage group length was 41.3 cM with a range from 32.7 cM (Chr7) to 58.7 cM (Chr5). The average marker density was 3.2 cM.

**Fig. 7:**
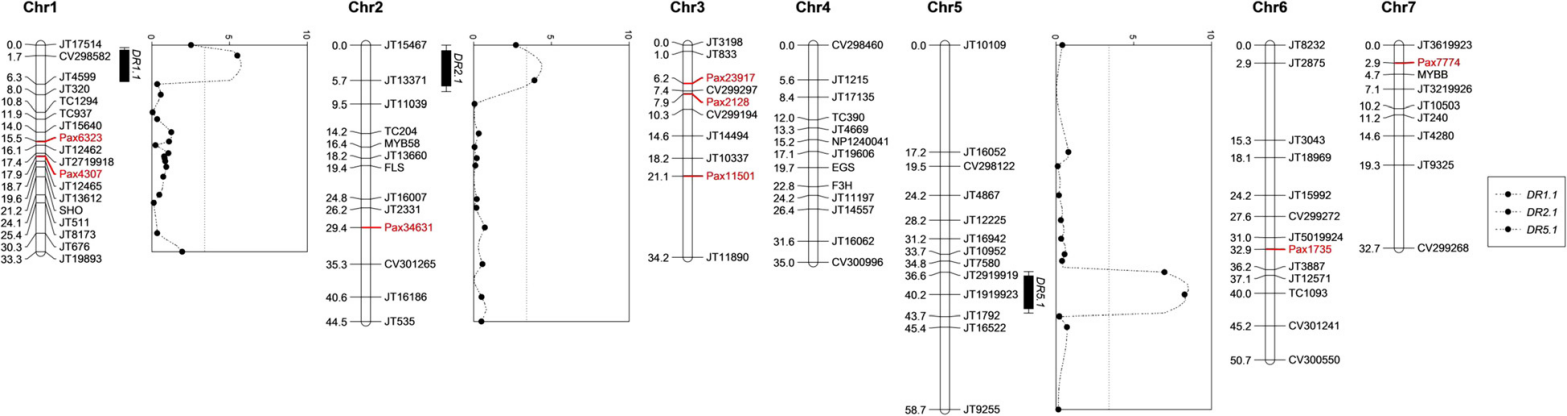
Genetic linkage map of a *P. integrifolia* × *P. axillaris* F_2_ population. Markers developed from plastochron-related genes are highlighted in red. QTL for development rate (DR) are indicated along with the corresponding LOD score along the linkage group

Similar to our previous study [11], three QTL for development rate located on chromosomes 1, 2 and 5 were detected. Together they explained 37 % of the variation for development rate (Fig. 7; Table 10). However, the QTL location on chromosome 1 shifted away from a CAPS marker developed from the isopentenyl transferase gene SHO, a gene originally identified in an activation-tagged line exhibiting increased lateral shoot production [67]. We found that although markers developed from plastochron-related genes were located on the same chromosomes as two of the QTL, they did not co-localize with development rate QTL regions. Linkage maps with higher resolution will help better understand the mechanisms for this trait.

**Table 10:** Summary of QTL for development rate (DRate; in nodes day^−1^) in a *P. integrifolia* × *P. axillaris* F_2_ population

Trait	LOD threshold	QTL	LG	Nearest marker	Position (cM)	LOD	a^a^	%VE^b^
DRate	3.4	DR1.1	1	CV298582	1.73	5.73	−0.03	11.3
		DR2.1	2	JT13371	4.00	4.36	−0.03	8.4
		DR5.1	5	JT1919923	39.59	8.51	0.05	17.5

^a^Additive effect of the *P. integrifolia* allele

^b^Percentage of variation explained

## Conclusions

High quality transcriptome assemblies were generated by RNA-seq data from three *Petunia* spp. and these were mined for molecular markers and plastochron-related transcripts. We used a SNP discovery pipeline to identify over 80,000 SNPs across three species. As the majority of the SNPs were located in exons and over 80 % of the SNP-containing transcripts can be annotated, these SNPs could possess utility in future gene discovery and breeding applications. Thirteen percent of the identified SNPs were polymorphic among all three species, and can be further used for comparative genomics and population diversity studies. Additionally, over 10,000 SNPs were transformed into CAPS markers and an additional set of SSR markers were developed. Plastochron-related transcripts were identified, converted to CAPS markers, and genetically mapped in a *P. integrifolia* × *P. axillaris* F_2_ population. Overall, these data provide sequence, expression information and a large marker resource for the *Petunia* community.

## Methods

### Plant material and growth conditions

Seeds of *Petunia axillaris* (PI667515) and *Petunia exserta* (OPGC943) were obtained from the Germplasm Resources Information Network. Seeds of *Petunia integrifolia* were purchased from Diane’s Flower Seeds (Ogdon, UT). Five different tissue types were harvested for RNAseq: 1) whole seedlings, 2) callus, 3) shoot apices, 4) flowers of mixed developmental stages, and 5) trichomes. For whole seedlings, *P. axillaris*, *P. integrifolia* and *P. exserta* seeds were sown on 100 mm diameter Petri plates containing half-strength Murashige and Skoog (MS) media. Plates were left unsealed to allow air exchange and grown under a 16-h photoperiod provided by fluorescent lamps (ca. 75 μmol m^−2^ s^−1^) at 22 °C. The date when seeds began to germinate was recorded (generally 2–3 d after sowing) and seedlings where harvested 21 d after this date. At harvest, media was removed from the roots, and plants were placed in 1.5 ml tubes and immediately frozen with liquid nitrogen.

For callus generation, seeds of each species were sterilized by: 1) soaking in sterilized water for 30 min, 2) rinsing with 90 % ethanol, 3) soaking in 50 % bleach for 7 min and 4) rinsing with sterilized water. Twenty seeds per species were sown in a magenta box containing 55 ml media [2.22 g MS, 15 g sucrose and 6 g agar per liter] and germinated under a 16 h photoperiod provided by fluorescent lamps (ca. 30 μmol m^−2^ s^−1^) at 22 °C. When seedlings reached a sufficient size, 1 cm^2^ leaf pieces were excised and placed onto 100 × 15 mm plates containing 25 ml callus-induction media [4.44 g MS, 100 μg 2,4-D, 20 g sucrose, and 8 g agar per liter] (Rao et al., 1973) and incubated in the dark at room temperature. Callus developed after approximately four weeks. Every few weeks until harvest, callus pieces were transferred to new plates containing the same media.

For the collection of shoot apices, flowers and trichomes, seed was sown in 288-cell plug trays in a soilless media and placed under in a greenhouse under intermittent mist at 24 °C. After emergence of the first true leaf, seedlings were removed from mist and placed in a glass-covered greenhouse at 20 °C under a 16 h photoperiod under ambient light, supplemented with 60 μmol m^−2^ s^−1^ provided by high-pressure sodium lamps. Shoot apices were collected when plants had unfolded 6–8 true leaves. Small leaves were removed with forceps to minimize contamination with leaf petiole tissue. The shoot apices from ca. 100 plants of each species were excised, placed in 15 ml conical tubes, and immediately frozen in liquid nitrogen. Flowers were harvested when a full range of reproductive stages of development were present, ranging from flower buds ca. 5 mm in length through the beginning fruit development. Harvested flowers were placed in paper envelopes, and frozen in liquid nitrogen. In order to harvest trichomes, stems and petioles from flowering Petunia plants were cut into ca. 5 cm pieces and immediately frozen in liquid nitrogen. The frozen tissue pieces were held using chilled forceps and the trichomes were removed from the stems by gentle scraping with a frozen paint brush and were collected in a mortar containing liquid nitrogen. Trichomes were immediately ground and transferred to 2 ml crew cap tubes for storage at −80 °C.

### RNA extraction

Total RNA was extracted from each tissue sample using the Trizol® reagent (LifeTechnologies) following the manufacturer’s instructions. Forty micrograms of RNA was treated for DNA contamination using RNase-free DNase set (Qiagen). DNase-treated RNA was purified using the RNeasy® MinElute Cleanup kit (Qiagen). RNA yield and quality were determined using gel electrophoresis, spectroscopy and the Agilent 2100 BioAnalyzer RNA 6000 Pico chip with RIN values ≥ 8.0 achieved (Agilent Technologies).

### RNA-seq library construction, sequencing, and analysis

A TruSeq RNA Sample Preparation kit (Illumina) was used to construct the cDNA libraries for sequencing. Illumina barcodes allowed pooling of 7–8 libraries per lane. Sequencing was completed on the Illumina HiSeq 2500 next-generation sequencer to 100 nt paired-end at the Michigan State University Research Technology Support Facility (RTSF; http://rtsf.msu.edu/; East Lansing, MI). Raw read quality was assessed with FastQC (http://www.bioinformatics.babraham.ac.uk/ projects/fastqc/). Sequences were filtered and trimmed based on quality metrics and adapter sequences were removed with TrimmomaticPE [68]. The TrimmomaticPE options employed included ILLUMINACLIP, SLIDINGWINDOW:5:20, LEADING:5, TRAILING:5, and HEADCROP:10–14. TrimmomaticPE outputs sequences into paired-end and single-end files requiring no further mate-specific filtering. Cleaned reads were reassessed with FastQC for quality visualization to ensure no further filtering was required.

### *De novo* assembly and expression analysis

Reads meeting quality standards were *de novo* assembled using the Velvet (version 1.1.07) and Oases (version 0.2.08) packages [69, 70]. K-mer lengths of 55, 57, 59, 61, 63, 65, 67, and 69 were tested and metrics collected for each assembly. Criterion including the total number of transcripts produced, Velvet N50 length, average transcript length, and average transcript read coverage were considered. A k-mer of 61 was selected representing the best balance of metrics for all assemblies. Two *de novo* assemblies were completed for each *Petunia* transcriptome to reduce redundancy and limit the number of raw reads confounding assemblies. Reads from the callus, shoot apex and seedling libraries composed the first *de novo* assembly. Resulting transcripts were concatenated into a single pseudomolecule or ‘artificial chromosome one’ to serve as our core tissue reference transcriptome. Reads from the trichome and flower libraries were then mapped to artificial chromosome one with Bowtie (version 1.0.0) and TopHat (version 1.4.1) [46]. Reads aligning to the artificial chromosome were discarded to eliminate redundancy of data already assembled. All unmapped reads from the flower and trichome libraries were then combined with the original callus, shoot apex, and seedling reads in a second *de novo* transcriptome assembly [42]; Supplemental Fig. 1). Transcripts from the second and final assembly were filtered for low complexity. Representative transcripts, comprised of the longest isoform were extracted from the final assemblies. Completeness of the assemblies was assessed by mapping all reads from each tissue library back to their respective *de novo* assembly (per species) individually with Bowtie and TopHat [46]. CEGMA or Core Eukaryotic Genes Mapping Approach [43], a pipeline used to accurately annotate core genes in eukaryotic genomes, was also searched to determine the quality and completeness of the assemblies. Reads from each tissue library were mapped back to the *de novo* assembly with Bowtie and TopHat. Estimation of transcript abundance was then completed with Cufflinks [71, 72]. Cufflinks reports expression abundance in Fragments Per Kilobase of exon model per Million fragments mapped, or FPKM. Values consider the number of reads supporting each model and counts are normalized.

### Functional annotation and identification of orthologous and paralogous gene families

Putative functions were assigned using BLASTX queries against the *Arabidopsis thaliana* protein database (TAIR10; http://www.arabidopsis.org/), UniRef100 (UniProt Knowledgebase Reference Clusters; release 63; http://www.uniprot.org/), RefSeq protein database (NCBI Reference Sequence Database; www.ncbi.nlm.nih.gov/refseq/), Pfam domains (version 27.0; pfam.xfam.org), and the NCBI non-redundant protein database (nr; www.ncbi.nlm.nih.gov/protein). BLASTX queries against all databases were searched using an e-value cutoff of 1e-5 and allowing a single hit. Hits were then processed accepting only those with > 30 % coverage and > 70 % identity to the subject. A total of 126 *P. axillaris,* 12 *P. integrifolia,* and two *P. exserta* representative transcripts were removed as suspected contamination based on their annotation. Functional annotation of the transcripts was also queried using web-based FastAnnotator [73]. FastAnnotator facilitates the integration of the annotation tools Blast2GO, PRIAM, and RPS BLAST, providing gene ontology classifications (GO), enzyme and domain identification. Transcripts were translated in batch using OrfPredictor [74] and orthologous and paralogous proteins in the three species were assigned with OrthoMCL v2.0.2 with default parameters and an e-value cut-off of 1e-10 [50].

### SNP Identification

Sequence reads from each tissue type were pooled for each species and mapped to the *Petunia axillaris* transcriptome using the Burrows-Wheeler Aligner (BWA) program [75] with default values. Duplicate reads were removed after the initial alignment, to eliminate reads that mapped to the same position of the transcriptome. Duplicate removal was performed for the aligned reads using picardTools/1.89 (broadinstitute.github.io/picard). The subsequent local realignment to correct misalignments due to the presence of indels was performed using the Genome Analysis Toolkit (GATK) [51] with the IndelRealigner function. Bam files for each genotype were compressed using ReduceReads from GATK. The initial variant calling was performed by HaplotypeCaller from GATK with a Phred-scaled confidence threshold of 30. After the initial variant calling, SNPs were filtered based on several criteria. The first round of SNP filtering employed the following steps: exclude three SNPs within 100 bp; filter out variants with zero mapping quality constituting more than 10 % of all reads at that site; exclude SNPs covered only by sequences on the same strand with an FS value (Phred-scaled p-value using Fisher’s exact test to detect strand bias) >60; exclude SNPs with a minor allele frequency <0.01; and exclude SNPs with low QD (quality by depth), low quality (<11), and low total depth of coverage (<11) by the default parameters recommended from GATK best practice with the VariantFiltration tool [53, 76]. The second round of SNP filtering was performed by custom Python (Python 2.7.8) [77] scripts with the following criteria: SNPs within the beginning and end 30 bp of the reference transcripts were excluded; SNPs were selected with at least two genotypes having a depth of coverage greater than 10; exclude loci with a heterozygous genotype call in *Petunia axillari*s. The intron-exon boundaries were predicted by aligning the *Petunia axillaris* transcripts to the draft *Petunia axillaris* genome scaffold sequences with GMAP [78]. SNPs within 30 bp of the exon-intron boundary region were then filtered out. Indels were not called because alternative splicing may impede reliable indel discovery. A custom Python script was used to extract the 5ʹ-UTR and 3ʹ-UTR and to calculate the distribution of SNPs in these regions. Depth of coverage was calculated by BEDTools [79]. Annotated unigenes containing SNPs were visualized by BGI WEGO (web gene ontology annotation plotting) (http://wego.genomics.org.cn/cgi-bin/wego/index.pl). KEGG pathways were assigned to unigenes containing SNPs using the online KEGG Automatic Annotation Server (KAAS) (http://www.genome.jp/tools/kaas/) [80]. KEGG Orthology (KO) assignment was applied using Bi-directional Best Hit (BBH) method with plant organisms (*Arabidopsis thaliana* (thale cress), *Arabidopsis lyrata* (lyrate rockcress), *Theobroma cacao* (cacao), *Glycine max* (soybean), *Fragaria vesca* (woodland strawberry), *Vitis vinifera* (wine grape), *Solanum lycopersicum* (tomato), and *Oryza sativa* L. ssp. *japonica* (Japanese rice) (RefSeq)) as references.

### SNP validation

A total of 55 SNPs were selected for validation. Primers were designed by first choosing SNPs where at least two species exhibited polymorphism. Unigenes with exons greater than 600 bp were then selected, SNPs or alleles in the sequences were masked using IUBI./IUPAC nucleotide acid code. Batch Primer3 (http://probes.pw.usda.gov/cgi-bin/batchprimer3/batchprimer3.cgi) was used for primer design using the “SNP flanking primers design” option. The expected amplicon sizes ranged from 250 to 350 bp, with primer sizes ranging from 18 to 27 bp, and primer T_m_ ranging from 57 to 63 °C. Genomic DNA was extracted using the DNeasy plant mini kit (QIAGEN, Valencia, CA, USA). PCR amplification of genomic DNA was carried out in a 10 μl reaction containing 1 × PCR buffer, 0.2 μl 10 μM dNTPs, 0.6 U of DNA polymerase, and 5 ng template DNA. The following PCR profile was used: an initial denaturation at 95 °C for 30 s, followed by 11 cycles of 95 °C for 30 s, 65 °C to 55 °C for 30 s, with a decrease of 1 °C per cycle, and 72 °C for 30 s, followed 30 cycles of 95 °C for 30 s, 58 °C for 30 s, and 72 °C for 30 s, followed by a final extension at 72 °C for 5 min. Amplicons were purified by Agencourt AMPure XP beads (Beckman Coulter, Pasadena, CA) and quantified by BioDrop Duo (Isogen Life Science, the Netherlands). Sanger sequencing of the amplicons was performed on an ABI 3730xl platform (Life Technologies, Carlsbad, CA) at the Michigan State University Genomics Core Facility. Sequences amplified from the same primer set were initially aligned with CLC Sequence Viewer 6.9.1 (CLC Bio, Valencia, CA, USA) for preliminary SNP verification. Sequence chromatograms were visualized by Seq Scanner 2 (Life Technologies, Carlsbad, CA) for further SNP confirmation.

### CAPS and SSR marker design

SNP markers were transformed into CAPS markers using the following pipeline. First, the SNP output was transformed into VCF (variant call format) format. Then, by using the previous GMAP output as reference, a new VCF file was generated by transforming the current SNP locations and genotypes to corresponding SNP locations and variant calls in the *P. axillaris* draft genome scaffolds by a custom Python script. Primers were designed with the galaxy-pcr-markers pipeline (https://github.com/cfljam/galaxy-pcr-markers) with the modification of producing only one pair of primers, minimum amplicon size of 200 bp and maximum amplicon size of 400 bp. A total of 20 commonly used restriction enzymes: *Alu*I, *Apa*I, *Bam*HI*, Bbr*PI, *Bfr*I, *Cla*I, *Dde*I, *Dpn*II, *Dra*I, *Eco*RI, *Hae*III, *Hin*cII, *Hin*fI, *Hpa*I, *Pvu*II, *Rsa*I, *Sac*I, *Sau*3AI, *Sma*I, and *Taq*I were selected for the pipeline. The unigene dataset were used for SSR identification with MISA (microsatellite identification tool) (http://pgrc.ipk-gatersleben.de/misa/). The SSR identification criteria were 6, 5, 5, 5, 5 repeats for di-, tri-, tetra-, penta-, and hexanucleotides, respectively. Primer pairs were designed from primer3_core (Primer3 2.3.6) (http://primer3.sourceforge.net/releases.php). Primer parameters were: designated amplicon size 100–280 bp, primer annealing temperature 55 to 65 °C, primer length 18 to 28 bp, and GC content from 45 to 55 %. Unigenes with homology to known genes involved in specifying plant development rate [16–18, 20, 25] were searched for possible CAPS markers from the entire CAPS output. The CAPS markers were used to genotype the three species by following the previous PCR protocol. CAPS markers were digested by the above mentioned restriction enzymes (New England Biolabs, Beverly, MA) and separated on 2 % NuSieve GTG agarose (Lonza, Basel, Switzerland) plus 1 % UltraPure agarose (Life Technologies, Carlsbad, CA) gel with 100 V for 2 h at room temperature.

### Candidate gene mapping and QTL mapping of development rate in an interspecific hybrid *Petunia* population

An interspecific hybrid F_2_ population developed from a cross between *P. integrifolia* and *P. axillaris* containing 164 individuals was used to identify QTL for development rate. Population development, the measurement of development rate, and the SSR-based genetic linkage map were reported previously [11]. CAPS markers developed from *P. axillaris* plastochron-related transcripts based on SNPs between *P. integrifolia* and *P. axillaris* were used to screen the F_2_ population. If no CAPS were previously designed from our SNP set, CAPS markers were manually designed with CAPS Designer (http://solgenomics.net/tools/caps_designer/caps_input.pl) and Primer3Plus (http://www.bioinformatics.nl/cgi-bin/primer3plus/primer3plus.cgi/). The updated genetic linkage map was generated with JoinMap 4.0 [81] with the Kosambi mapping function [82]. The recombination threshold was set to 0.3 and the logarithmic odds (LOD) score was set to 5. The locus order was calculated by the regression module of JoinMap4. Linkage group numbers were assigned according to the previous study [11]. QTL mapping was conducted with MapQTL 6 [83]. A permutation test with 1000 permutations was used to establish the LOD significance threshold. The initial QTL mapping was performed by interval mapping (a single-QTL model). Multiple QTL model mapping (MQM) was then used to reduce the background noise. Significant QTL and regions were graphically visualized using MapChart 2.1 [84].

### Accession numbers

Sequences are available under Genbank/EMBL/DDBJ BioProject numbers PRJNA262254, PRJNA262142, and PRJNA261953. These include SRA (Sequence Read Archive) runs for *P. axillaris*: SRR1585615, SRR1585635, SRR1585830, and SRR1585954-1585955; *P. exserta*: SRR1586492-1586494, SRR1586500, and SRR1586504; *P. integrifolia*: SRR1587109, SRR1587150-1587151, and SRR1587153-1587154. Transcriptome Shotgun Assembly projects were also deposited under accessions GBRU00000000 (*P. axillaris*), GBRT00000000 (*P. exserta*), and GBRV00000000 (*P. integrifolia*). The versions described in this paper are the first versions of these assemblies (GBRU01000000, GBRT01000000, GBRV01000000). The SNPs are available at NCBI dbSNP under accession numbers ss1750993386 - ss1751108187.

## Additional files

Additional file 1: Figure S1. Work flow used for assemblies allowing incorporation of reads from all 5 tissue libraries while removing redundancy. **Figure S2.** Distributions of the number of isoforms for each transcript. **Figure S3.** SNP discovery pipeline with reads aligned to *P. axillaris* reference transcriptome assembly. **Figure S4.** Gene Ontology (GO) categories of the unigenes and unigenes with SNPs. **Figure S5.** Gene Ontology (GO) categories of the unigenes with more than ten SNPs. Distribution of the GO categories assigned to the unigenes were annotated in three categories: cellular components, molecular functions, and biological processes. **Table S1.** Primer sequences used for SNP validation by Sanger sequencing. **Table S2.** Primer sequences for CAPS markers developed from plastochron-related genes. (DOCX 782 kb) Additional file 2:
**Supplemental Dataset 1.** Petunia unigene locus identifiers, expression levels, annotation and sequences. (XLSX 11680 kb) Additional file 3:
**Supplemental Dataset 2.** OrthoMCL clusters across three *Petunia* species. (XLSM 1433 kb) Additional file 4:
**Supplemental Dataset 3.** Gene Ontology annotations of representative transcripts. (XLS 16720 kb) Additional file 5:
**Supplemental Dataset 4.** CAPS markers developed from SNPs. (XLSX 1499 kb) Additional file 6:
**Supplemental Dataset 5.** SSR primers derived from ***P. axillaris*** **transcriptome.** (XLSX 414 kb) Additional file 7:
**Supplemental Dataset 6.** SSR validation results. (XLSX 13 kb)
