# Supplementary material for: Transcriptome-enabled marker discovery and mapping of plastochron-related genes in Petunia spp
Source: BMC Genomics. 2015 Sep 24;16(1):726. doi: 10.1186/s12864-015-1931-4 (PMC4581106; doi:10.1186/s12864-015-1931-4)
Supplement: Additional file 1: Figure S1. — Work flow used for assemblies allowing incorporation of reads from all 5 tissue libraries while removing redundancy. Figure S2. Distributions of the number of isoforms for each transcript. Figure S3. SNP discovery pipeline with reads aligned to P. axillaris reference transcriptome assembly. Figure S4. Gene Ontology (GO) categories of the unigenes and unigenes with SNPs. Figure S5. Gene Ontology (GO) categories of the unigenes with more than ten SNPs. Distribution of the GO categories assigned to the unigenes were annotated in three categories: cellular components, molecular functions, and biological processes. Table S1. Primer sequences used for SNP validation by Sanger sequencing. Table S2. Primer sequences for CAPS markers developed from plastochron-related genes. (DOCX 782 kb) [file 12864_2015_1931_MOESM1_ESM.docx]

Artificial chromosome #1

Callus, meristem, and seedling reads

*De novo* assembly #1

Flower and trichome

reads mapped

Annotation

Reads from each library mapped

Callus, meristem and seedling reads used for assembly #1

*De novo* assembly #2

Artificial chromosome #2

Calculation of expression abundances (FPKM)

Reads not aligning/Novel reads

(From flower and trichome libraries)

Reads that align

***De Novo* assembly #2**

**Annotation**

**Artificial chromosome #2**

**Calculation of expression abundances (FPKM)**

**Reads from each**

**library mapped**

Figure 2. Assembly and expression analysis work flow used for the ‘total’ *P. axillaris*, *P. exserta* and *P. integrifolia* transcriptomes.

**Figure S1**. Work flow used for assemblies allowing incorporation of reads from all 5 tissue libraries while removing redundancy.

Figure S2. Distributions of the number of isoforms for each transcript.

**Figure S3**. SNP discovery pipeline with reads aligned to *P. axillaris* reference transcriptome assembly.


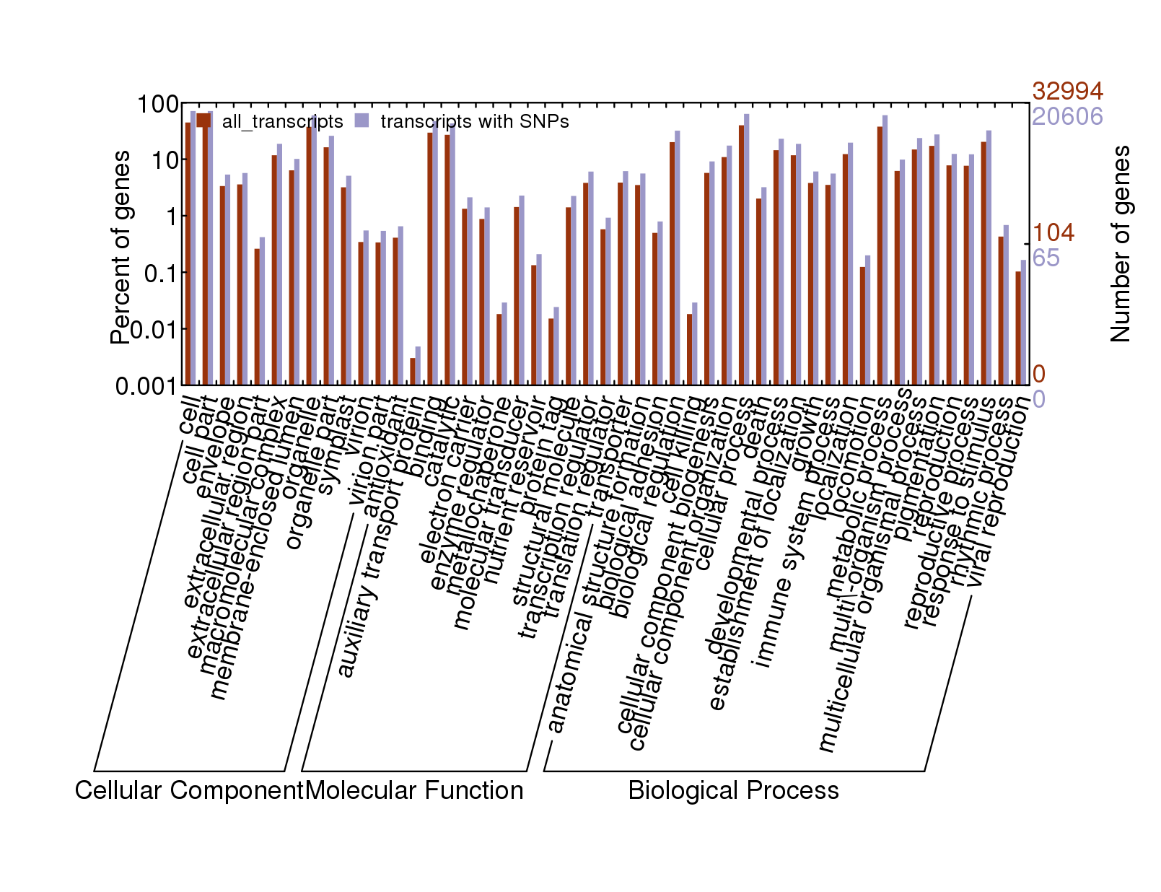


**Figure S4**. Gene Ontology (GO) categories of the unigenes and unigenes with SNPs. Distribution of the GO categories assigned to the unigenes were annotated in three categories: cellular components, molecular functions, and biological processes.


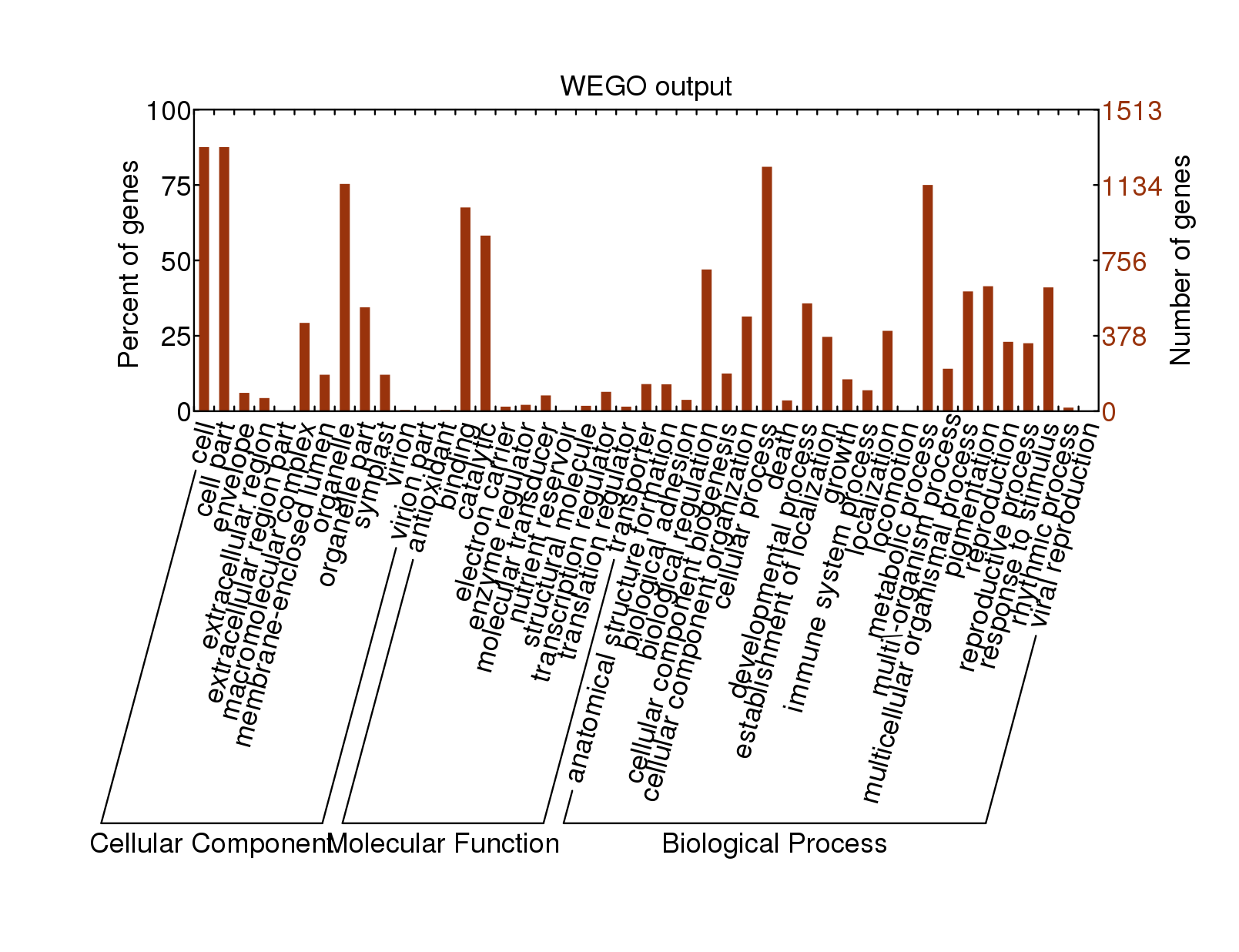


**Figure S5.** Gene Ontology (GO) categories of the unigenes with more than ten SNPs. Distribution of the GO categories assigned to the unigenes were annotated in three categories: cellular components, molecular functions, and biological processes.

|  | Primer name | *Petunia axillaris* Unigene name | SNP position | Primer sequence |
| --- | --- | --- | --- | --- |
| 1 | V_Locus_4442_F | Locus_4442_Transcript_14/22_Confidence_0.191_Length_3860 | 2496 | TTGGGGGAATTTAGGGTTTC |
| 2 | V_Locus_4442_R | Locus_4442_Transcript_14/22_Confidence_0.191_Length_3860 | 2496 | GCAAAGGGTAGTCAGGCAAA |
| 3 | V_Locus_4493_F | Locus_4493_Transcript_4/5_Confidence_0.615_Length_2780 | 1620 | CCAGATGCCGAAACCTTAAA |
| 4 | V_Locus_4493_R | Locus_4493_Transcript_4/5_Confidence_0.615_Length_2780 | 1620 | ACGAAGCAGCTCCACAAGAT |
| 5 | V_Locus_4955_F | Locus_4955_Transcript_2/4_Confidence_0.667_Length_1169 | 417 | TCCAGCTGAAAACACCTTCA |
| 6 | v_Locus_4955_R | Locus_4955_Transcript_2/4_Confidence_0.667_Length_1169 | 417 | TGCAATACGACCATCAGCAT |
| 7 | V_Locus_5011_F | Locus_5011_Transcript_6/7_Confidence_0.467_Length_4097 | 985 | GCTCCTGAACAGGGTTTGTC |
| 8 | V_Locus_5011_R | Locus_5011_Transcript_6/7_Confidence_0.467_Length_4097 | 985 | TGGTACCTGGAACTGCATCA |
| 9 | V_Locus_5128_F | Locus_5128_Transcript_1/1_Confidence_1.000_Length_8389 | 3775 | CATCGTCCGATGAGCATAAA |
| 10 | V_Locus_5128_R | Locus_5128_Transcript_1/1_Confidence_1.000_Length_8389 | 3775 | GGGGAGGTTCATGGTTTCTT |
| 11 | V_Locus_5244_F | Locus_5244_Transcript_5/6_Confidence_0.643_Length_5153 | 2995 | GCCACAACTTCATCAGCAAG |
| 12 | V_Locus_5244_R | Locus_5244_Transcript_5/6_Confidence_0.643_Length_5153 | 2995 | TGGGATTTTTGCAAGAGGTT |
| 13 | V_Locus_5665_F | Locus_5665_Transcript_1/1_Confidence_1.000_Length_2440 | 1404 | TGGCTTCCGGATGAAAATAG |
| 14 | V_Locus_5665_R | Locus_5665_Transcript_1/1_Confidence_1.000_Length_2440 | 1404 | TGAATGGTCTTGTCAACCAAA |
| 15 | V_Locus_6193_F | Locus_6193_Transcript_5/5_Confidence_0.667_Length_4558 | 2635 | CAAGGCCGATGACAAGAAAA |
| 16 | V_Locus_6193_R | Locus_6193_Transcript_5/5_Confidence_0.667_Length_4558 | 2635 | GGAGCAGGTGTAGGCTTTTG |
| 17 | V_Locus_6590_F | Locus_6590_Transcript_4/5_Confidence_0.714_Length_2397 | 373 | CATCGGGTTCGGAATACTTG |
| 18 | V_Locus_6590_R | Locus_6590_Transcript_4/5_Confidence_0.714_Length_2397 | 373 | GGAAAAATGGGACATTACGC |
| 19 | V_Locus_6907_F | Locus_6907_Transcript_4/4_Confidence_0.625_Length_3310 | 1366 | TCAGTGCAAAGTCCCATTTG |
| 20 | V_Locus_6907_R | Locus_6907_Transcript_4/4_Confidence_0.625_Length_3310 | 1366 | TGGAAATTAGGGTGCATGTG |
| 21 | V_Locus_6970_F | Locus_6970_Transcript_6/6_Confidence_0.643_Length_2915 | 1796 | GACAATTTGCAGAGCCCACT |
| 22 | V_Locus_6970_R | Locus_6970_Transcript_6/6_Confidence_0.643_Length_2915 | 1796 | AAATTCACCACCACCAGGAA |
| 23 | V_Locus_7169_F | Locus_7169_Transcript_1/1_Confidence_1.000_Length_1778 | 461 | ATCGTCTCCCGAAACTTCCT |
| 24 | V_Locus_7169_R | Locus_7169_Transcript_1/1_Confidence_1.000_Length_1778 | 461 | GAAAGCCAAAAATGCAGCAG |
| 25 | V_Locus_7225_F | Locus_7225_Transcript_9/10_Confidence_0.458_Length_3974 | 2129 | AACACCATCCAAAATGGAACA |
| 26 | V_Locus_7225_R | Locus_7225_Transcript_9/10_Confidence_0.458_Length_3974 | 2129 | GAAGCATCTTGCAATCAGCA |
| 27 | V_Locus_7293_F | Locus_7293_Transcript_3/4_Confidence_0.625_Length_2999 | 310 | TCTTTTGCATTCCCTCTCTG |
| 28 | V_Locus_7293_R | Locus_7293_Transcript_3/4_Confidence_0.625_Length_2999 | 310 | CCAACACTGCACACAATTCA |
| 29 | V_Locus_8156_F | Locus_8156_Transcript_1/1_Confidence_1.000_Length_817 | 402 | GAGGCGGAAATTGATCGTAA |
| 30 | V_Locus_8156_R | Locus_8156_Transcript_1/1_Confidence_1.000_Length_817 | 402 | TTTTTCCAATTTCCAAGCTCA |
| 31 | V_Locus_8764_F | Locus_8764_Transcript_3/5_Confidence_0.615_Length_2955 | 812 | CACCCTTTGTCCCAATCTAAG |
| 32 | V_Locus_8764_R | Locus_8764_Transcript_3/5_Confidence_0.615_Length_2955 | 812 | TCCAGCCCCTCCTTTTTATT |
| 33 | V_Locus_9135_F | Locus_9135_Transcript_1/1_Confidence_1.000_Length_2393 | 863 | CAACAACATTCCTTCATCATCA |
| 34 | V_Locus_9135_R | Locus_9135_Transcript_1/1_Confidence_1.000_Length_2393 | 863 | CCCCACTTTGTTGACTTGGT |
| 35 | V_Locus_9276_F | Locus_9276_Transcript_1/1_Confidence_1.000_Length_1898 | 1425 | CTTCACGCCTAACTGAGCTG |
| 36 | V_Locus_9276_R | Locus_9276_Transcript_1/1_Confidence_1.000_Length_1898 | 1425 | GGACTTCCACACCATGTCCT |
| 37 | V_Locus_9589_F | Locus_9589_Transcript_1/1_Confidence_1.000_Length_3088 | 1425 | TTTGGTCCCATCGTCAATTT |
| 38 | V_Locus_9589_R | Locus_9589_Transcript_1/1_Confidence_1.000_Length_3088 | 1425 | TGTTTGGAGCATTTGTTGGA |
| 39 | V_Locus_9778_F | Locus_9778_Transcript_3/4_Confidence_0.667_Length_4071 | 3465 | TATGCATGGATTACGCTGCT |
| 40 | V_Locus_9778_R | Locus_9778_Transcript_3/4_Confidence_0.667_Length_4071 | 3465 | GAAGGAATAGGGGCGGTAAG |
| 41 | V_Locus_10068_F | Locus_10068_Transcript_7/10_Confidence_0.380_Length_4003 | 2207 | AATCCCCGTCTTTCAGGAAC |
| 42 | V_Locus_10068_R | Locus_10068_Transcript_7/10_Confidence_0.380_Length_4003 | 2207 | CGAATCACCACCAAGGTTTC |
| 43 | V_Locus_10804_F | Locus_10804_Transcript_6/6_Confidence_0.611_Length_2053 | 1014 | TTGACTGGTGGCACTGTTCT |
| 44 | V_Locus_10804_R | Locus_10804_Transcript_6/6_Confidence_0.611_Length_2053 | 1014 | GCTAAATGAAGCAGCGGAAG |
| 45 | V_Locus_11475_F | Locus_11475_Transcript_1/1_Confidence_1.000_Length_3121 | 2086 | AAGCAGGGGTATAGCATCCA |
| 46 | V_Locus_11475_R | Locus_11475_Transcript_1/1_Confidence_1.000_Length_3121 | 2086 | TAAGGGGAAAAACCATGAGC |
| 47 | V_Locus_12184_F | Locus_12184_Transcript_2/8_Confidence_0.579_Length_2441 | 971 | CCTCCCTGCAAAACATTCAT |
| 48 | V_Locus_12184_R | Locus_12184_Transcript_2/8_Confidence_0.579_Length_2441 | 971 | CCTCTCTCCTCCCTGGTCTT |
| 49 | V_Locus_12561_F | Locus_12561_Transcript_4/6_Confidence_0.647_Length_2250 | 1315 | CCCTGTCCACCTTTTCAATG |
| 50 | V_Locus_12561_R | Locus_12561_Transcript_4/6_Confidence_0.647_Length_2250 | 1315 | GATGTGGATGCCGAATTTCT |
| 51 | V_Locus_13610_F | Locus_13610_Transcript_5/6_Confidence_0.688_Length_1938 | 1303 | AGGGGACACTGATCCAAGAG |
| 52 | V_Locus_13610_R | Locus_13610_Transcript_5/6_Confidence_0.688_Length_1938 | 1303 | GCAATTTGCACACAACAAGC |
| 53 | V_Locus_13631_F | Locus_13631_Transcript_7/8_Confidence_0.414_Length_2572 | 1107 | CAATGTTGGGAAAGTAACTGGA |
| 54 | V_Locus_13631_R | Locus_13631_Transcript_7/8_Confidence_0.414_Length_2572 | 1107 | AACATACCGCCTTTGCTCAG |
| 55 | V_Locus_13652_F | Locus_13652_Transcript_2/4_Confidence_0.600_Length_3106 | 1636 | TTCCTTCAGTTGGAGAATTGG |
| 56 | V_Locus_13652_R | Locus_13652_Transcript_2/4_Confidence_0.600_Length_3106 | 1636 | GGAGCTCGTGAAGAACTGCT |
| 57 | V_Locus_13922_F | Locus_13922_Transcript_5/8_Confidence_0.560_Length_1829 | 753 | TCACCATCCCTCATTTCCAT |
| 58 | V_Locus_13922_R | Locus_13922_Transcript_5/8_Confidence_0.560_Length_1829 | 753 | TTTTTCAAAGGGACGGAACA |
| 59 | V_Locus_14239_F | Locus_14239_Transcript_1/1_Confidence_1.000_Length_3442 | 1427 | CAAATGCAGGTTTGCTGTCA |
| 60 | V_Locus_14239_R | Locus_14239_Transcript_1/1_Confidence_1.000_Length_3442 | 1427 | CCAGCTTTCTTTGAGGCAAC |
| 61 | P_404_F | Locus_404_Transcript_2/4_Confidence_0.625_Length_2043 | 1638 | GATGCTGGGCAGGAATTTAT |
| 62 | P_404_R | Locus_404_Transcript_2/4_Confidence_0.625_Length_2043 | 1638 | TGGCCAAACAAGAAATTTGA |
| 63 | P_560_F | Locus_560_Transcript_2/9_Confidence_0.704_Length_3640 | 2850 | CCGCATGGATGGTAGGTAAC |
| 64 | P_560_R | Locus_560_Transcript_2/9_Confidence_0.704_Length_3640 | 2850 | AGAACGATGCACGAGTTCAC |
| 65 | P_587_F | Locus_587_Transcript_4/9_Confidence_0.690_Length_7892 | 7535 | CACCCTCCAAAAATCCCATA |
| 66 | P_587_R | Locus_587_Transcript_4/9_Confidence_0.690_Length_7892 | 7535 | TTTAGCCTGTTTGGGTGAGC |
| 67 | P_841_F | Locus_841_Transcript_1/2_Confidence_0.750_Length_2089 | 483 | TTCATTGGAGGAATGGAAGG |
| 68 | P_841_R | Locus_841_Transcript_1/2_Confidence_0.750_Length_2089 | 483 | ACTTAGCGCCATACGGACAT |
| 69 | P_1379_F | Locus_1379_Transcript_1/1_Confidence_1.000_Length_1789 | 1035 | GCCACAACTTCCATGAAACC |
| 70 | P_1379_R | Locus_1379_Transcript_1/1_Confidence_1.000_Length_1789 | 1035 | CAGCAGCTCGTCGATTACAA |
| 71 | P_1400_F | Locus_1400_Transcript_1/3_Confidence_0.667_Length_1585 | 799 | CTGCTCGTGCAAACAACAAT |
| 72 | P_1400_R | Locus_1400_Transcript_1/3_Confidence_0.667_Length_1585 | 799 | TGTGGTGGAGTCCCTTCTTC |
| 73 | P_1485_F | Locus_1485_Transcript_1/2_Confidence_1.000_Length_2427 | 928 | TGGGACATCATCCACAAAAA |
| 74 | P_1485_R | Locus_1485_Transcript_1/2_Confidence_1.000_Length_2427 | 928 | TTTAAGCAGGCGAGTTGGTC |
| 75 | P_2713_F | Locus_2713_Transcript_1/1_Confidence_1.000_Length_2288 | 1872 | TTTCTCATTCCAACCACAACC |
| 76 | P_2713_R | Locus_2713_Transcript_1/1_Confidence_1.000_Length_2288 | 1872 | GGCCTTTTGTTTCCCAAAAT |
| 77 | P_3046_F | Locus_3046_Transcript_1/1_Confidence_1.000_Length_4433 | 3835 | TCCTTGGGAAATCCCTTTTC |
| 78 | P_3046_R | Locus_3046_Transcript_1/1_Confidence_1.000_Length_4433 | 3835 | ACCAAGATATTGGCGAATGC |
| 79 | P_3260_F | Locus_3260_Transcript_8/10_Confidence_0.655_Length_4253 | 1152 | GCTCAATTCCAAGGAACCAA |
| 80 | P_3260_R | Locus_3260_Transcript_8/10_Confidence_0.655_Length_4253 | 1152 | CTCCCTCCAGCAATGAGTTC |
| 81 | P_4136_F | Locus_4136_Transcript_1/6_Confidence_0.385_Length_3340 | 1054 | CCCTGATGTGCTTCCAAACT |
| 82 | P_4136_R | Locus_4136_Transcript_1/6_Confidence_0.385_Length_3340 | 1054 | AAGGTGGAAAAGCAACTGGA |
| 83 | P_4252_F | Locus_4252_Transcript_1/1_Confidence_1.000_Length_1682 | 563 | AGCCCAATCTTTTCCCCATA |
| 84 | P_4252_R | Locus_4252_Transcript_1/1_Confidence_1.000_Length_1682 | 563 | GTGACACAACGTCTGCTGCT |
| 85 | P_4439_F | Locus_4439_Transcript_1/1_Confidence_1.000_Length_1584 | 576 | GCCTACAAATCACAGGCACA |
| 86 | P_4439_R | Locus_4439_Transcript_1/1_Confidence_1.000_Length_1584 | 576 | CCAGGCTCAGTGGCTTACAT |
| 87 | P_1960_F | Locus_1960_Transcript_1/2_Confidence_0.750_Length_4367 | 1233 | AATCACATGGCATGAGGTGA |
| 88 | P_1960_R | Locus_1960_Transcript_1/2_Confidence_0.750_Length_4367 | 1233 | TTGCTCAGGATCGGTAGGTT |
| 89 | P_2011_F | Locus_2011_Transcript_2/3_Confidence_0.333_Length_3731 | 1554 | ATACGCAACGCAAGCCTTAT |
| 90 | P_2011_R | Locus_2011_Transcript_2/3_Confidence_0.333_Length_3731 | 1554 | AATGAAGGAGGCAGTCATGG |
| 91 | P_2053_F | Locus_2053_Transcript_3/6_Confidence_0.688_Length_3798 | 1576 | GGGGCATTTTGATTTTTGAA |
| 92 | P_2053_R | Locus_2053_Transcript_3/6_Confidence_0.688_Length_3798 | 1576 | AGACCCCGAAAGGTGAGAGT |
| 93 | P_4219_F | Locus_4219_Transcript_7/10_Confidence_0.581_Length_4920 | 633 | CCTAAGCTTGGCAAAGGAAA |
| 94 | P_4219_R | Locus_4219_Transcript_7/10_Confidence_0.581_Length_4920 | 633 | TCCATCCAGATGAAGCTCAG |
| 95 | P_4440_F | Locus_4440_Transcript_1/2_Confidence_1.000_Length_2844 | 1692 | TCTGTGAGGCAGCTTGAATG |
| 96 | P_4440_R | Locus_4440_Transcript_1/2_Confidence_1.000_Length_2844 | 1692 | CCGGAACCCATAAAGTTCAA |
| 97 | P_5868_F | Locus_5868_Transcript_4/5_Confidence_0.500_Length_3236 | 734 | CAGTGCTGAATGAGCAGGAA |
| 98 | P_5868_R | Locus_5868_Transcript_4/5_Confidence_0.500_Length_3236 | 734 | GGAGTGCAGAAAGGAAATGG |
| 99 | P_8450_F | Locus_8450_Transcript_1/2_Confidence_0.750_Length_3636 | 2962 | GAGCACCATTCGAAAAGCTC |
| 100 | P_8450_R | Locus_8450_Transcript_1/2_Confidence_0.750_Length_3636 | 2962 | TTCTGAATCGGCGGTTTTT |

**Table S1**. Primer sequences used for SNP validation by Sanger sequencing.

| Marker name | *Petunia axillaris* transcript | Forward sequence | Reverse sequence | Restriction enzyme |
| --- | --- | --- | --- | --- |
| Pax7774 | Locus_7774_Transcript_1/1_Confidence_1.000_Length_3771 | TCTGAAGGCGAGGAAGATCC | GTTGAAGTCTGTGAGGCTGG | TaqI |
| Pax28305 | Locus_28305_Transcript_3/3_Confidence_0.600_Length_4234 | GAGTGGAAAAGGCTGGTCCA | ACTTTTTCTCAAGTTTGGTGAGGT | AflII |
| Pax17737 | Locus_17737_Transcript_3/3_Confidence_0.667_Length_3550 | CCGTCCAGGAAGGTGAAG | TAGCCTATGGTGCCCAAAAC | Hpy188III |
| Pax17054 | Locus_17054_Transcript_3/3_Confidence_0.600_Length_2365 | AGTGAGGCCAAACAAGCTTG | TTGGTGGAGGCCATGATTATG | AluI |
| Pax4307 | Locus_4307_Transcript_1/3_Confidence_0.667_Length_2763 | GGTACCACGAAGACCAACGA | GGCTCTTCAACCAAAGATAATAGCA | AluI |
| Pax2898 | Locus_2898_Transcript_1/1_Confidence_1.000_Length_2484 | CCACCTCGAGATGGCAAGTT | TGTCTTCTCATGCTTTTCACCA | TaqI |
| Pax2128 | Locus_2128_Transcript_1/2_Confidence_0.833_Length_3063 | GCCACATCAAATGAGGAGAAA | CAATCTGCTTTGGTGCATGT | MaeIII |
| Pax23917 | Locus_23917_Transcript_1/1_Confidence_1.000_Length_2956 | TCCAGCTCACCTTACTTCCC | TCCTTTGCCAACTTCTGTGC | TaqI |
| Pax34631 | Locus_34631_Transcript_2/2_Confidence_0.750_Length_962 | GAGATGGCATGTCAATCGGG | GGGCATCAGTCTGAATGCAG | AluI |
| Pax1735 | Locus_1735_Transcript_5/6_Confidence_0.538_Length_4646 | CCTCTTCATGTTGCAGCTGG | TTCCATGCTTCAACTGCCAC | RsaI |
| Pax11501 | Locus_11501_Transcript_1/4_Confidence_0.625_Length_1639 | ATGGGCTCAGCAACTCAGAA | CGGAGAAGAAGACCCAGATG | HinfI |
| Pax30395 | Locus_30395_Transcript_1/2_Confidence_0.750_Length_1427 | CCAAATACTAGAGAATCCGAGCA | AGGGTCGGTTCCTTGATTCC | AluI |
| Pax6323 | Locus_6323_Transcript_2/3_Confidence_0.667_Length_3369 | GTTCCTCGATCGGCAATTCC | ACTCAGGGATCTCAAGCCTG | AluI |

**Table S2**. Primer sequences for CAPS markers developed from plastochron-related genes.
